# Aberrant DNA methylation results in altered gene expression in non-alcoholic steatohepatitis-related hepatocellular carcinomas

**DOI:** 10.1007/s00432-020-03298-4

**Published:** 2020-07-19

**Authors:** Ying Tian, Eri Arai, Satomi Makiuchi, Noboru Tsuda, Junko Kuramoto, Kentaro Ohara, Yoriko Takahashi, Nanako Ito, Hidenori Ojima, Nobuyoshi Hiraoka, Masahiro Gotoh, Teruhiko Yoshida, Yae Kanai

**Affiliations:** 1 Department of Pathology, Keio University School of Medicine, 35 Shinanomachi, Shinjuku-ku, Tokyo, 160-8582 Japan; 2 Bioscience Department, Solution Knowledge Center, Mitsui Knowledge Industry Co., Ltd, Tokyo, 105-6215 Japan; 3 Pathology Division, Department of Pathology and Clinical Laboratories, National Cancer Center Hospital, Tokyo, 104-0045 Japan; 4 Fundamental Innovative Oncology Core Center, National Cancer Center Research Institute, Tokyo, 104-0045 Japan

**Keywords:** DNA methylation, Expression alteration, Non-alcoholic steatohepatitis, Hepatocellular carcinoma, *DCAF4L2*

## Abstract

**Purpose:**

The aim of this study was to investigate DNA methylation alterations in non-alcoholic steatohepatitis (NASH)-related hepatocellular carcinomas (HCCs).

**Methods:**

Genome-wide DNA methylation analysis was performed using the Infinium Human Methylation 450 K BeadChip, and levels of mRNA expression were analyzed by quantitative reverse transcription-PCR.

**Results:**

Compared to 36 samples of normal control liver tissue (C), DNA methylation alterations were observed on 19,281 probes in 22 samples of cancerous tissue (T) obtained from patients showing histological features compatible with NASH in their non-cancerous liver tissue (N). Among those probes, 1396 were located within CpG islands or their shores and shelves, designed around the transcription start sites of 726 genes. In representative genes, such as *DCAF4L2*, *CKLF*, *TRIM4*, *PRC1*, *UBE2C* and *TUBA1B*, both DNA hypomethylation and mRNA overexpression were observed in T samples relative to C samples, and the levels of DNA methylation and mRNA expression were inversely correlated with each other. DNA hypomethylation occurred even in N samples at the precancerous NASH stage, and this was inherited by or further strengthened in T samples. DNA hypomethylation of *DCAF4L2*,* CKLF* and *UBE2C* was observed in both NASH-related and viral hepatitis-related HCCs, whereas that of *TRIM4*,* PRC1* and *TUBA1B* occurred in a NASH-related HCC-specific manner. DNA hypomethylation and/or mRNA overexpression of these genes was frequently associated with the necroinflammatory grade of NASH and was correlated with poorer tumor differentiation.

**Conclusion:**

DNA methylation alterations may occur under the necroinflammatory conditions characteristic of NASH and participate in NASH-related hepatocarcinogenesis through aberrant expression of tumor-related genes.

**Electronic supplementary material:**

The online version of this article (10.1007/s00432-020-03298-4) contains supplementary material, which is available to authorized users.

## Introduction

Non-alcoholic steatohepatitis (NASH), a hepatic manifestation of metabolic syndrome (Bessone et al. 2019) resulting in the development of liver cirrhosis, has shown an alarming increase in recent years. Although hepatitis B virus (HBV) or hepatitis C virus (HCV) infection followed by chronic hepatitis and liver cirrhosis used to be the main cause of hepatocellular carcinoma (HCC), there is evidence that NASH is becoming another precancerous condition for HCC (Kim and El-Serag 2019). However, the molecular background of NASH-related HCC is not yet fully understood.

It is well known that not only genomic, but also epigenomic alterations participate in carcinogenesis in various human organs (Baylin and Jones 2016; Jones et al. 2016). Epigenome alterations have also attracted a great deal of attention as the molecular basis of metabolic disorders (Keating and El-Osta 2015). In the light of these facts, it would be informative to understand the significance of DNA methylation alterations in NASH-related hepatocarcinogenesis. Due to the difficulty in obtaining appropriate human samples, many previous studies of NASH-related HCC have been conducted using animal models (Borowa-Mazgaj et al. 2019). Even in studies using human clinical samples, DNA methylation status has been examined only for individual tumor-related genes (Tian et al. 2015), and studies employing genome-wide DNA methylation analysis have been very limited.

Recently, single-CpG-resolution genome-wide DNA methylation screening using the Infinium assay (Bibikova et al. 2009) has been introduced for analysis of various human tissue specimens (Tsumura et al. 2019; Makabe et al. 2019). In the context of NASH, a previous Infinium analysis using human tissue specimens has focused merely on the differences between NASH and simple steatosis (de Mello et al. 2017). Although we have also reported the results of Infinium assay using pathological tissue specimens, we focused mainly on DNA methylation profiles at the precancerous stages: such profiles in NASH were compared with those of chronic hepatitis and liver cirrhosis in patients with HBV or HCV infection, and those in patients without HCCs were compared with those of patients with HCCs (Kuramoto et al. 2017). Although a study of HCCs by The Cancer Genome Atlas (TCGA) employed the Infinium assay (https://portal.gdc.cancer.gov/legacy-archive/files/d794c956-9333-488d-b784-aaaf86c2e5de), the background etiology of only 3% of deposited HCC samples was “non-alcoholic fatty liver disease”. Thus, as the severity of steatosis in such samples was unclear, it was not possible to confirm whether or not such HCCs were truly derived from NASH (Ally et al. 2017). On overview of the currently available literature therefore suggests that the genome-wide DNA methylation profiles of NASH-related HCCs themselves have yet to be reported in detail.

In the present study, we employed the Infinium assay for genome-wide DNA methylation (methylome) analysis and quantitative reverse transcription (RT)-PCR for analysis of gene expression in samples of NASH-related human HCC and identified tumor-related genes possibly participating in the development of NASH-related HCCs. We then examined in meticulous detail the correlations between DNA methylation status and the expression levels of identified genes, as well as the various clinicopathological parameters of both NASH and HCC.

## Materials and methods

### Patients and tissue samples

For the present analysis, we used 22 paired samples of non-cancerous liver tissue (N) and corresponding cancerous tissue (T) obtained by partial hepatectomy from 22 HCC patients whose N samples showed histological features compatible with NASH. All 22 patients were negative for both HBV surface antigen and anti-HCV antibody. NASH stage was evaluated microscopically on the basis of the histological scoring system for NASH (Kleiner et al. 2005) and the Brunt classification (Brunt et al. 1999). Histological diagnosis of HCCs was made in accordance with the World Health Organization classification (Hirohashi et al. 2000) and the tumor–node–metastasis (TNM) classification (Brierley et al. 2017). The age, sex and clinicopathological characteristics of the subjects are summarized in Supplementary Table S1A. For comparison, 36 samples of normal control liver tissue (C), obtained by partial hepatectomy from 36 patients with liver metastases of primary colorectal cancers without HBV or HCV infection, chronic hepatitis, liver cirrhosis or HCC, were examined. In addition, 36 samples of HCCs from patients with HBV and/or HCV infection (Supplementary Table S1B) were analyzed.

None of the patients had received preoperative treatment, and all underwent surgery at the National Cancer Center Hospital, Tokyo, Japan. Immediately after surgical removal, tissue specimens were frozen and stored in liquid nitrogen at the National Cancer Center Biobank, Tokyo, Japan, until use in research, in accordance with the Japanese Society of Pathology Guidelines for the handling of pathological tissue samples for genomic research (Kanai et al. 2018). This study was approved by the Ethics Committees of the National Cancer Center, Tokyo, Japan, and Keio University, and was performed in accordance with the Declaration of Helsinki. All of the patients provided written informed consent prior to inclusion of their specimens in the study.

### Infinium assay

High-molecular-weight DNA was extracted from fresh-frozen tissue samples using phenol–chloroform, followed by dialysis. Five hundred nanograms of genomic DNA was subjected to bisulfite treatment using an EZ DNA Methylation-Gold™ Kit (Zymo Research, Irvine, CA), in accordance with the manufacturer’s protocol. DNA methylation status at 485,577 CpG loci was examined at single-CpG resolution using the Infinium HumanMethylation450 BeadChip (Illumina, San Diego, CA) (Bibikova et al. 2009). After hybridization, the specifically hybridized DNA was fluorescence labeled by a single-base extension reaction and detected using an iScan reader (Illumina) in accordance with the manufacturer’s protocol.

The data were then assembled using GenomeStudio methylation software (Illumina). At each CpG site, the ratio of the fluorescence signal was measured using a methylated probe relative to the sum of the methylated and unmethylated probes, i.e., the so-called β-value, which ranges from 0.00 to 1.00, reflecting the methylation level of an individual CpG site. Some of the results of the Infinium assay were used in our previous study focusing on comparison with viral hepatitis-related HCCs (Kuramoto et al. 2017) and deposited in the GEO database (https://www.ncbi.nlm.nih.gov/geo/, Accession number GSE89852).

### Gene ontology (GO) enrichment analysis

A network topology-based analysis was conducted using the WEB-based GEne SeT AnaLysis Toolkit (Webgestalt, https://www.webgestalt.org) and the network expansion method (Ashburner et al. 2000), with reference to the BioGRID 3.5 database (https://www.thebiogrid.org) and setting the number of top-ranking neighbors to 10. The generated network was investigated for enriched GO biological processes.

### Real-time quantitative RT-PCR analysis

Total RNA was isolated from all 22 paired N and T samples and 31 C samples, for which sufficient amounts of tissue were available for RNA extraction even after extraction of genomic DNA for the Infinium assay, using TRIzol reagent (Life Technologies, Carlsbad, CA). cDNA was generated from total RNA using random primers and SuperScript IV Reverse Transcriptase (Invitrogen, Carlsbad. CA). Levels of expression of mRNA were determined using the PowerUp SYBR Green Master Mix (Applied Biosystems, Foster City, CA) on the 7500 Fast Real-Time PCR system (Applied Biosystems) employing the relative standard curve method. PCR primers were designed using the Primer Designer software (Thermo Fisher Scientific, UK, https://www.thermofisher.com/order/genome-database/), Primer-BLAST software (https://www.ncbi.nlm.nih.gov/tools/primer-blast/index.cgi) or by reference to a previous paper (Li et al. 2016). The sequences of the PCR primer sets employed are shown in Supplementary Table S2. Experiments were performed in triplicate, and the mean value for the three experiments was used as the threshold cycle (Ct) value. All Ct values were normalized to that of the *GAPDH* gene in the same sample.

### Statistics

In the Infinium assay, the call proportions (*P* < 0.01 for detection of signals above the background) for 681 probes (shown in Supplementary Table S3) in all of the examined tissue samples were less than 90%. Since such a low proportion may due to polymorphism at the probe of CpG sites, these 681 probes were excluded from subsequent analysis, as described previously (Ohara et al. 2017). Meanwhile, the missing data rate defined as the percentage of missing values across all of the tissue samples was also taken into consideration, 45 probes for which more than 10% of the data were missing being thus removed from the analysis. In addition, probes on chromosomes X and Y were removed to avoid any gender-specific methylation bias, leaving a final total of 470,429 autosomal CpG sites.

Differences in levels of DNA methylation and mRNA expression between C and T samples were examined by Welch’s *t* test. Correlations between mRNA expression levels or DNA methylation status in T samples on the one hand, and clinicopathological parameters on the other were tested by Fisher's exact test. Statistical analyses were performed using the SPSS program version 24 (IBM Corp, Armonk, NY) and programing language R. Differences at *P* values of less than 0.05 were considered statistically significant.

## Results

### DNA methylation alterations in T samples relative to C samples

The Infinium assay identified 19,281 probes that exhibited aberrant DNA methylation in T samples relative to C samples (*P* < 0.01, Welch’s *t* test with Bonferroni correction, and a Δ*β*_T−C_ value of more than 0.2 or less than − 0.2). To identify genes with alterations of DNA methylation potentially resulting in alterations of expression, among the 19,281 probes, we focused on 7256 that were located within the CpG island based on the UCSC Genome Browser (https://genome.ucsc.edu/), N-Shelf (2000 bp region 5′ adjacent to N-Shore), N-Shore (2000 bp region 5′ adjacent to CpG island), S-Shore (2000 bp region 3′ adjacent to CpG island), and S-Shelf (2000 bp region 3′ adjacent to S-Shore). Among these 7256 probes, 1396 were located within the region from the transcription start site (TSS) to 1500 bp upstream of the specific gene. Among the 726 genes for which the 1396 probes were designed, 219 genes showed a significant inverse correlation between the levels of DNA methylation and mRNA expression (Pearson’s correlation coefficient *r* < − 0.25, *P* < 0.05), based on data for cancers derived from multiple organs, i.e. HCC, clear cell renal cell carcinoma, lung adenocarcinoma, stomach adenocarcinoma and endometrial carcinoma, deposited in The Cancer Genome Atlas (TCGA) database (https://cancergenome.nih.gov). These 219 genes are listed in Supplementary Table S4.

### GO enrichment analysis using aberrantly methylated genes in T samples

The 219 genes shown in Supplementary Table S4 were subjected to GO enrichment analysis. The top 20 significant biological processes are summarized in Table 1. The top process that was significant even after correction using the false discovery rate (FDR) was NIK (NF-KB [nuclear factor-kappa B] inducing kinase, also termed MAP3K14, mitogen-activated protein kinase kinase kinase 14)/NF-ΚB signaling, which reportedly has an essential role in the immune response and carcinogenesis (Valiño-Rivas et al. 2019). Furthermore, four and two processes in Table 1 were involved in the NF-ΚB signaling pathway (GO:0038061, GO:0007250, GO:1901222 and GO:1901224) and in the Wnt signaling pathway (GO:0060071 and GO:0090263), respectively.

**Table 1:** Gene Ontology enrichment analysis using the 219 genes aberrantly methylated in hepatocellular carcinomas and included in Supplementary Table S4

GO ID	Biological process^a^	*P*	False discovery rate	Genes aberrantly methylated in hepatocellular carcinomas and involved in the process
GO:0038061	NIK/NF-κB signaling	< 0.001	3.74 E−02	*RIPK3*, *PSMA8*, *ADIPOR1*, *PSMD2*, *TRAF2*, *CARD14*, *TNFRSF10A*, *PSMD6*
GO:0007250	Activation of NF-κB-inducing kinase activity	< 0.001	9.89 E−01	*TRAF2*, *CARD14*, *TNFRSF10A*
GO:1901222	Regulation of NIK/NF-κB signaling	< 0.001	9.89 E−01	*ADIPOR1*, *TRAF2*, *CARD14*, *TNFRSF10A*
GO:0032502	Developmental process	< 0.001	9.89.E−01	*SEMA4F*, *CFTR*, *RIPK3*, *SCLT1*, *PSMA8*, *ELAVL1*, *RBM24*, *NCOA6*, *PRAME*, *USP21*, *SALL3*, *SFN*, *HK2*, *HOXA10*, *HOXD8*, *APP*, *INPP5D*, *ITGA6*, *ITGA4*, *ITGA7*, *KRT19*, *LFNG*, *MEST*, *ASCL2*, *NEDD4*, *NTRK1*, *ADIPOR1*, *CCHCR1*, *POU5F1*, *PPP2R3A*, *PCDHB8*, *PSMD2*, *VANGL2*, *PTCH1*, *PTN*, *RPS6KA1*, *RRBP1*, *SCN8A*, *SPI1*, *UBE2V2*, *VIM*, *ZNF217*, *USP7*, *CAD*, *THOC6*, *LRRK1*, *HDAC11*, *FZD6*, *PARD6B*, *ZIC5*, *TNFRSF10A*, *CDKL2*, *INA*, *SPAG6*, *CD44*, *PSMD6*
GO:0048856	Anatomical structure development	< 0.001	9.89 E−01	*SEMA4F*, *CFTR*, *RIPK3*, *SCLT1*, *PSMA8*, *ELAVL1*, *RBM24*, *NCOA6*, *USP21*, *SALL3*, *SFN*, *HK2*, *HOXA10*, *HOXD8*, *APP*, *INPP5D*, *ITGA6*, *ITGA4*, *ITGA7*, *KRT19*, *LFNG*, *MEST*, *ASCL2*, *NEDD4*, *NTRK1*, *ADIPOR1*, *CCHCR1*, *POU5F1*, *PPP2R3A*, *PCDHB8*, *PSMD2*, *VANGL2*, *PTCH1*, *PTN*, *RPS6KA1*, *SCN8A*, *SPI1*, *UBE2V2*, *VIM*, *ZNF217*, *USP7*, *CAD*, *THOC6*, *LRRK1*, *HDAC11*, *FZD6*, *PARD6B*, *ZIC5*, *TNFRSF10A*, *INA*, *SPAG6*, *CD44*, *PSMD6*
GO:1990000	Amyloid fibril formation	< 0.001	9.89 E−01	*RIPK3*, *APP*
GO:0033209	Tumor necrosis factor-mediated signaling pathway	0.001	9.89 E−01	*PSMA8*, *PSMD2*, *TRAF2*, *CARD14*, *TNFRSF10A*, *PSMD6*
GO:0007275	Multicellular organism development	0.001	9.89 E−01	*SEMA4F*, *RIPK3*, *SCLT1*, *PSMA8*, *ELAVL1*, *NCOA6*, *USP21*, *SALL3*, *SFN*, *HK2*, *HOXA10*, *HOXD8*, *APP*, *INPP5D*, *ITGA6*, *ITGA4*, *ITGA7*, *KRT19*, *LFNG*, *ASCL2*, *NEDD4*, *NTRK1*, *ADIPOR1*, *CCHCR1*, *POU5F1*, *PPP2R3A*, *PCDHB8*, *PSMD2*, *VANGL2*, *PTCH1*, *PTN*, *RPS6KA1*, *SCN8A*, *SPI1*, *UBE2V2*, *VIM*, *ZNF217*, *USP7*, *CAD*, *THOC6*, *LRRK1*, *HDAC11*, *FZD6*, *PARD6B*, *ZIC5*, *TNFRSF10A*, *INA*, *CD44*, *PSMD6*
GO:0007166	Cell surface receptor signaling pathway	0.001	9.89 E−01	*SEMA4F*, *PSMA8*, *SALL3*, *CD274*, *HNRNPF*, *APP*, *INPP5D*, *ITGA6*, *ITGA4*, *ITGA7*, *KRT19*, *LFNG*, *MICB*, *NEDD4*, *NTRK1*, *ADIPOR1*, *RNF220*, *MAPK13*, *PMEPA1*, *PSMD2*, *VANGL2*, *PTCH1*, *PTN*, *TRAF2*, *VIM*, *CARD14*, *LRRK1*, *FZD6*, *TNFRSF10A*, *CD44*, *PSMD6*
GO:0060071	Wnt signaling pathway, planar cell polarity pathway	0.002	9.89 E−01	*PSMA8*, *PSMD2*, *VANGL2*, *FZD6*, *PSMD6*
GO:0090175	Regulation of establishment of planar polarity	0.002	9.89 E−01	*PSMA8*, *PSMD2*, *VANGL2*, *FZD6*, *PSMD6*
GO:1901224	Positive regulation of NIK/NF-κB signaling	0.002	9.89 E−01	*TRAF2*, *CARD14*, *TNFRSF10A*
GO:0009968	Negative regulation of signal transduction	0.002	9.89 E −01	*PSMA8*, *PRAME*, *MPV17L*, *SALL3*, *INPP5D*, *ITGA6*, *LFNG*, *NEDD4*, *ADIPOR1*, *PMEPA1*, *PSMD2*, *PTCH1*, *TRAF2*, *FZD6*, *TNFRSF10A*, *CD44*, *PSMD6*
GO:0035019	Somatic stem cell population maintenance	0.002	9.89 E−01	*ASCL2*, *POU5F1*, *VANGL2*, *SPI1*
GO:0090263	Positive regulation of canonical Wnt signaling pathway	0.002	9.89 E−01	*PSMA8*, *RNF220*, *PSMD2*, *LRRK1*, *PSMD6*
GO:0031398	Positive regulation of protein ubiquitination	0.002	9.89 E−01	*UBE2C*, *PSMA8*, *PSMD2*, *DERL1*, *TSPYL5*, *PSMD6*
GO:0001736	Establishment of planar polarity	0.002	9.89 E−01	*PSMA8*, *PSMD2*, *VANGL2*, *FZD6*, *PSMD6*
GO:0007164	Establishment of tissue polarity	0.002	9.89 E−01	*PSMA8*, *PSMD2*, *VANGL2*, *FZD6*, *PSMD6*
GO:0060965	Negative regulation of gene silencing by miRNA	0.002	9.89 E−01	*ELAVL1*, *POU5F1*
GO:0007165	Signal transduction	0.002	9.89 E−01	*SEMA4F*, *CFTR*, *RIPK3*, *PSMA8*, *NCOA6*, *ZDHHC17*, *PRAME*, *SH3BP1*, *MPV17L*, *SALL3*, *SFN*, *ANK1*, *CD274*, *HNRNPF*, *APP*, *INPP5D*, *ITGA6*, *ITGA4*, *ITGA7*, *KRT19*, *LFNG*, *MICB*, *NEDD4*, *NTRK1*, *ADIPOR1*, *RNF220*, *WSB2*, *MAPK13*, *PMEPA1*, *PSMD2*, *VANGL2*, *PTCH1*, *PTN*, *RPS6KA1*, *ARHGAP9*, *RASSF10*, *LYNX1*, *TRAF2*, *VIM*, *ZNF217*, *GGCT*, *CARD14*, *DERL1*, *LRRK1*, *FZD6*, *SPSB2*, *TSPYL5*, *TNFRSF10A*, *CDKL2*, *CD44*, *PSMD6*

^a^After omission of several processes related to specific organs other than the liver, e.g. establishment of body hair or bristle planar orientation (GO:0048104) and regulation of synapse structure or activity (GO:0050803), the top 20 processes are listed

### Identification of cancer-related genes showing overexpression possibly due to DNA hypomethylation in T samples relative to C samples

**Fig. 1:**
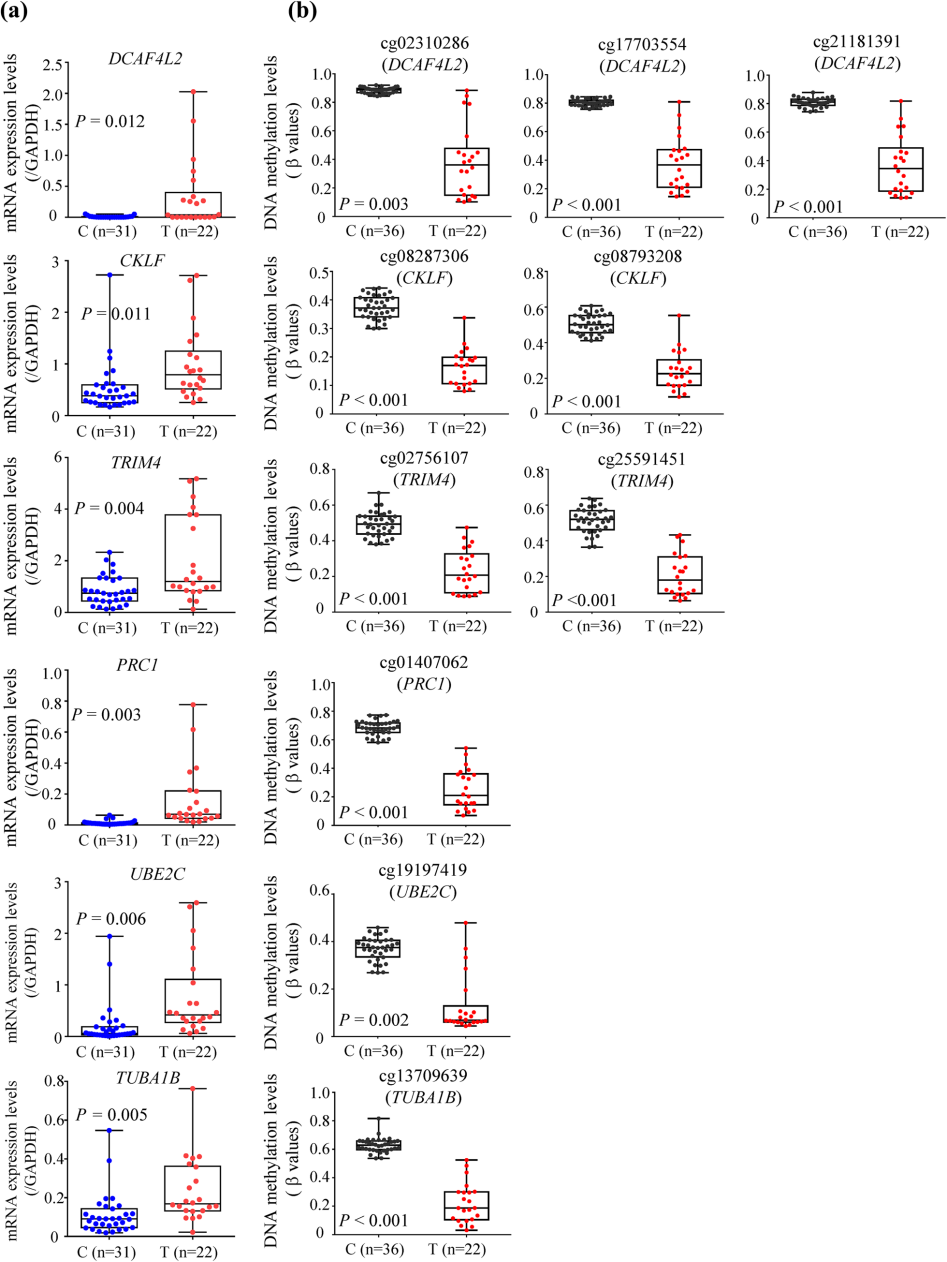
Scatterplot of the levels of mRNA expression (**a**) and DNA methylation (**b**) of the *DCAF4L2*, *CKLF*, *TRIM4*, *PRC1*, *UBE2C* and *TUBA1B* genes in samples of normal control liver tissue (C) and tumorous tissue (T). **a** mRNA expression levels were determined by SYBR green-based real-time quantitative PCR analysis. **b** DNA methylation levels were determined by Infinium assay. The Infinium probe ID for each gene is shown in parentheses. Levels of mRNA expression for all six genes were significantly increased in T samples (*n* = 22) relative to C samples (*n* = 31), whereas levels of DNA methylation on all ten probes designed for the six genes were significantly decreased in T samples (*n* = 22) relative to C samples (*n* = 36)

Taking into consideration literature-based implications in the process of carcinogenesis, including but not limited to hepatocarcinogenesis, we further focused on 24 genes that showed DNA hypomethylation in T samples and these are included in Supplementary Table S4. SYBR green-based real-time quantitative PCR analysis was performed for these 24 genes (Supplementary Table S2) using the 31 C and 22 T samples. This revealed that the levels of expression of mRNAs for the *DCAF4L2 *(*DDB1* and *CUL4 associated factor 4-like 2*), *CKLF* (*chemokine-like factor*),* TRIM4* (*tripartite motif-containing 4*),* PRC1* (*protein regulator of cytokinesis 1*),* UBE2C* (*ubiquitin conjugating enzyme E2 C*) and *TUBA1B* (*tubulin alpha 1b*) genes were significantly increased in T samples relative to C samples (*P* < 0.05) (Fig. 1). Using 75 tissue samples in our own cohort, i.e., 31 C, 22 N and 22 T samples, each gene actually showed a significant inverse correlation between the level of DNA methylation and that of mRNA expression (*r* < − 0.3 and *P* < 0.05) (Table 2).

**Table 2:** Genes showing significant inverse correlations between the levels of mRNA expression and DNA methylation in all of the 75 tissue samples, normal control liver tissue samples (*n* = 31 and *n* = 36 for mRNA expression and DNA methylation quantification, respectively), non-cancerous liver tissue samples (*n* = 22) obtained from patients with non-alcoholic steatohepatitis-related hepatocellular carcinomas, and the corresponding cancerous tissue samples (*n* = 22) in the present cohort

Gene	Average mRNA expression levels (/GAPDH, mean ± SD) (*n* = 75)	Probe ID^a^	Chromosome	Position^b^	Average DNA methylation levels (mean ± SD) (*n* = 80)	Correlation coefficient (*n* = 75)	*P*^c^ (*n* = 75)
*DCAF4L2*	0.112 ± 0.345	cg02310286	8	88,886,432	0.735 ± 0.259	− 0.613	< 0.001
		cg17703554	8	88,886,339	0.682 ± 0.220	− 0.563	< 0.001
		cg21181391	8	88,886,411	0.682 ± 0.231	− 0.576	< 0.001
*CKLF*	0.602 ± 0.547	cg08287306	16	66,585,924	0.298 ± 0.103	− 0.386	< 0.001
		cg08793208	16	66,585,449	0.405 ± 0.132	− 0.307	0.007
*TRIM4*	1.178 ± 1.100	cg02756107	7	99,517,460	0.415 ± 0.139	− 0.588	< 0.001
		cg25591451	7	99,517,509	0.416 ± 0.157	− 0.597	< 0.001
*PRC1*	0.060 ± 0.126	cg01407062	15	91,538,575	0.539 ± 0.201	− 0.486	< 0.001
*UBE2C*	0.335 ± 0.568	cg19197419	20	44,441,612	0.283 ± 0.126	− 0.336	0.003
*TUBA1B*	0.016 ± 0.013	cg13709639	12	49,526,040	0.485 ± 0.192	− 0.397	< 0.001

^a^Probe ID for the Infinium HumanMethylation450 BeadChip (Illumina)

^b^National Center for Biotechnology Information database (Genome Built 37)

^c^*P* values of less than 0.05 were considered statistically significant

**Fig. 2:**
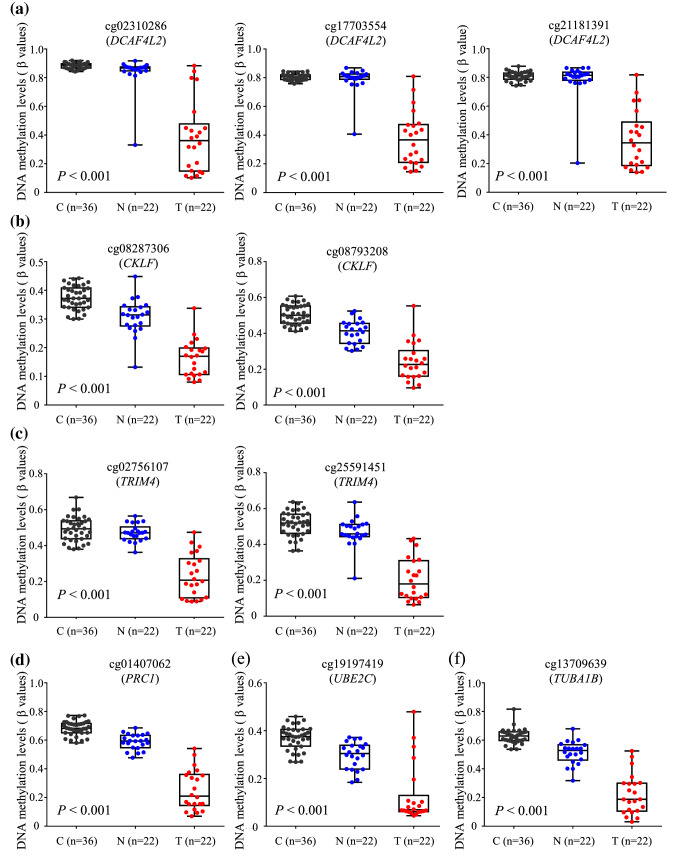
Ordered differences in levels of DNA methylation in normal control liver tissue (C) (*n* = 36) and non-cancerous liver tissue (N) (*n* = 22) obtained from patients with non-alcoholic steatohepatitis-related hepatocellular carcinomas relative to the corresponding tumorous tissue (T) samples (*n* = 22) for the *DCAF4L2* (**a**), *CKLF* (**b**), *TRIM4* (**c**), *PRC1* (**d**), *UBE2C* (**e**) and *TUBA1B* (**f**) genes. *P* values by Jonckheere–Terpstra trend test are shown in each panel. DNA methylation alterations of such genes in N samples relative to C samples were inherited by or further strengthened in the T samples themselves

Our previous study had provided evidence that DNA methylation alterations occurred even at the early and precancerous stage during multistage hepatocarcinogenesis (Kuramoto et al. 2017). We then subjected these six genes to the Jonckheere–Terpstra trend test using all 80 tissue samples including N samples that showed histological features compatible with NASH and were possibly at the precancerous stage. This revealed significant ordered differences in DNA methylation status from C (*n* = 36) to N (*n* = 22) and then to T (*n* = 22) samples (Fig. 2), indicating that the DNA hypomethylation of these six genes in N samples relative to C samples was inherited by or further strengthened in the T samples themselves. Ordered differences in mRNA expression levels including N samples are shown in Supplementary Figure S1.

**Fig. 3:**
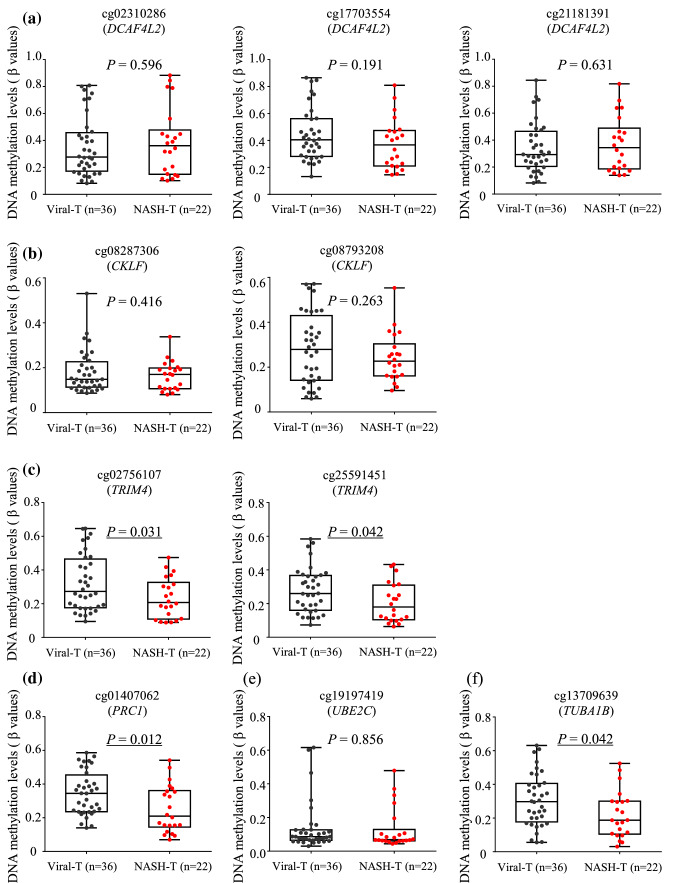
DNA methylation levels of the *DCAF4L2* (**a**), *CKLF* (**b**), *TRIM4* (**c**), *PRC1* (**d**), *UBE2C* (**e**) and *TUBA1B* (**f**) genes in samples of viral hepatitis-related hepatocellular carcinoma (HCC) (viral-T) (*n* = 36) and non-alcoholic steatohepatitis-related HCC (NASH-T) (*n* = 22). Levels of DNA methylation for the *DCAF4L2*, *CKLF* and *UBE2C* genes in NASH-T samples were not significantly different from those in viral-T samples, whereas levels of DNA methylation for the *TRIM4*, *PRC1* and *TUBA1B* genes in NASH-T samples were significantly lower than those in viral-T samples, indicating that DNA hypomethylation of the *TRIM4*, *PRC1* and *TUBA1B* genes had occurred specifically during NASH-related hepatocarcinogenesis. *P* values of < 0.05 are underlined

We then compared the DNA methylation levels of these six genes in the T samples (*n* = 22) with DNA methylation data for HCCs from patients with HBV and/or HCV infection (*n* = 36). The DNA methylation levels for *DCAF4L2*, *CKLF* and *UBE2C* in T samples were not significantly different from those in viral hepatitis-related HCCs, whereas the DNA methylation levels for *TRIM4*, *PRC1* and *TUBA1B* in T samples were significantly lower than those in viral hepatitis-related HCCs, indicating that DNA hypomethylation of the *TRIM4*, *PRC1* and *TUBA1B* genes occurred specifically during NASH-related hepatocarcinogenesis (Fig. 3).

### Clinicopathological impact of DNA hypomethylation and overexpression in T samples

With respect to each of the *DCAF4L2*, *CKLF*,* TRIM4*,* PRC1*,* UBE2C* and *TUBA1B* genes, 22 T samples were divided into high- and low-DNA methylation groups using DNA methylation levels at the 25th percentile as cutoff points based on the results of Infinium assay. We examined correlations between high or low DNA methylation levels in T samples on the one hand, and clinicopathological parameters of N samples (steatosis, lobular inflammation, hepatocellular ballooning, non-alcoholic fatty liver disease activity score [NAS] (Kleiner et al. 2005), necroinflammatory grade and stage based on the Brunt classification (Brunt et al. 1999)) and of T samples (tumor differentiation (Hirohashi et al. 2000), portal vein involvement and pathological TNM stage (Brierley et al. 2017)) on the other. Lower levels of DNA methylation on the three Infinium probes (cg02310286, cg17703554 and cg21181391) designed for the *DCAF4L2* gene, those on cg08287306 designed for the *CKLF* gene, those on cg25591451 designed for the *TRIM4* gene and those on cg13709639 designed for the *TUBA1B* gene were significantly correlated with a higher NAS score and an advanced necroinflammatory grade in N samples (*P* = 0.043) (Table 3).

**Table 3:** Correlations between DNA methylation levels, defined as low or high using the 25th percentile methylation levels as cutoff values, for the *DCAF4L2*, *CKLF*, *TRIM4*, *PRC1*, *UBE2C* and *TUBA1B* genes and the clinicopathological parameters of patients with non-alcoholic steatohepatitis-related hepatocellular carcinomas (HCCs)

Clinicopathological parameters	Number of patients	*DCAF4L2* (cg02310286)				*DCAF4L2* (cg17703554)			*DCAF4L2 *(cg21181391)			*CKLF* (cg08287306)			*CKLF* (cg08793208)			*TRIM4* (cg02756107)			*TRIM4* (cg25591451)			*PRC1* (cg01407062)			*UBE2C* (cg19197419)			*TUBA1B* (cg13709639)		
		Low	High		*P*	Low	High	*P*	Low	High	*P*	Low	High	*P*	Low	High	*P*	Low	High	*P*	Low	High	*P*	Low	High	*P*	Low	High	*P*	Low	High	*P*
Histological findings in non-cancerous liver tissue																																
Steatosis (%)^a^																																
5–33	15	3	12		0.316	2	13	0.132	2	13	0.132	3	12	0.316	3	12	0.689	3	12	0.316	2	13	0.132	2	13	0.132	3	12	0.316	2	13	0.132
33–66	6	1	5			2	4		2	4		1	5		2	4		1	5		2	4		2	4		1	5		2	4	
> 66	1	1	0			1	0		1	0		1	0		0	1		1	0		1	0		1	0		1	0		1	0	
Lobular inflammation (foci/field)^a^																																
< 2	17	2	15		0.055	3	14	0.548	2	15	0.055	3	14	0.548	5	12	0.290	3	14	0.548	3	14	0.548	3	14	0.548	4	13	1.000	3	14	0.548
2–4	5	3	2			2	3		3	2		2	3		0	5		2	3		2	3		2	3		1	4		2	3	
Ballooning^a^																																
Few	19	3	16		0.117	3	16	0.117	3	16	0.117	3	16	0.117	4	15	1.000	4	15	1.000	3	16	0.117	4	15	1.000	4	15	1.000	3	16	0.117
Many	3	2	1			2	1		2	1		2	1		1	2		1	2		2	1		1	2		1	2		2	1	
Nonalcoholic fatty liver disease activity score^a^																																
3–5	20	3	17	*0.043*		3	17	*0.043*	3	17	*0.043*	3	17	*0.043*	5	15	1.000	4	16	0.411	3	17	*0.043*	4	16	0.411	4	16	0.411	3	17	*0.043*
6–7	2	2	0			2	0		2	0		2	0		0	2		1	1		2	0		1	1		1	1		2	0	
Necroinflammatory grade^b^																																
Mild to moderate	20	3	17		*0.043*	3	17	*0.043*	3	17	*0.043*	3	17	*0.043*	5	15	1.000	4	16	0.411	3	17	*0.043*	4	16	0.411	4	16	0.411	3	17	*0.043*
Severe	2	2	0			2	0		2	0		2	0		0	2		1	1		2	0		1	1		1	1		2	0	
Brunt stage^c^																																
1	5	0	5		0.630	1	4	1.000	1	4	0.886	1	4	1.000	0	5	0.254	1	4	0.886	1	4	0.886	2	3	0.886	2	3	0.630	3	2	0.185
2	10	3	7			3	7		2	8		3	7		2	8		2	8		2	8		2	8		3	7		1	9	
3	5	2	3			1	4		2	3		1	4		2	3		1	4		1	4		1	4		0	5		1	4	
4	2	0	2			0	2		0	2		0	2		1	1		1	1		1	1		0	2		0	2		0	2	
Clinicopathological characteristics of HCCs																																
Differentiation^d^																																
Well	4	0	4		0.157	2	2	0.222	1	3	0.529	1	3	1.000	0	4	0.108	2	2	0.222	2	2	0.222	1	3	0.529	1	3	1.000	0	4	0.783
Moderately	13	5	8			3	10		4	9		3	10		2	11		3	10		3	10		4	9		3	10		4	9	
Poorly	5	0	5			0	5		0	5		1	4		3	2		0	5		0	5		0	5		1	4		1	4	
Portal vein invasion																																
Negative	10	1	9		0.323	2	8	1.000	2	8	1.000	2	8	1.000	3	7	0.624	3	7	0.624	2	8	1.000	3	7	0.624	2	8	1.000	2	8	1.000
Positive	12	4	8			3	9		3	9		3	9		2	10		2	10		3	9		2	10		3	9		3	9	
Pathological tumor–node–metastasis stage^e^																																
IA–IB	8	1	7		0.613	1	7	0.613	2	6	1.000	1	7	0.613	3	5	0.309	3	5	0.309	2	6	1.000	2	6	1.000	1	7	0.613	1	7	0.613
II–IIIB	14	4	10			4	10		3	11		4	10		2	12		2	12		3	11		3	11		4	10		4	10	

^a^According to Kleiner et al. (2005)

^b^According to the Brunt classification (1999)

^c^Used for assessment of hepatic fibrosis (Brunt et al. 1999)

^d^In accordance with the World Health Organization classification (Hirohashi et al. 2000)

^e^In accordance with the Union for International Cancer Control (UICC) classification (Brierley et al. 2017). *P* values of < 0.05 are in italics

On the basis of the results of quantitative RT-PCR analysis, 22 T samples were divided into high- and low-expression groups using mRNA expression levels at the 75th percentile as cutoff points. Correlations between high or low levels of mRNA expression in T samples on the one hand and clinicopathological parameters of N and T samples on the other were examined. Higher expression of the *DCAF4L2* and *CKLF* genes was significantly correlated with a higher NAS score and an advanced necroinflammatory grade (*P* = 0.043) in N samples (Table 4).

**Table 4:** Correlations between mRNA expression levels, defined as low or high using 75th percentile methylation levels as cutoff values, for the *DCAF4L2*, *CKLF*, *TRIM4*, *PRC1*, *UBE2C* and *TUBA1B* genes and clinicopathological parameters of patients with non-alcoholic steatohepatitis-related hepatocellular carcinomas (HCCs)

Clinicopathological parameters	Number of patients	*DCAF4L2*			*CKLF*			*TRIM4*			*PRC1*			*UBE2C*			*TUBA1B*		
		High	Low	*P*	High	Low	*P*	High	Low	*P*	High	Low	*P*	High	Low	*P*	High	Low	*P*
Histological findings in non-cancerous liver tissue																			
Steatosis (%)^a^																			
5–33	15	3	12	0.316	2	13	0.132	4	11	1.000	4	11	1.000	3	12	0.689	3	12	0.689
33–66	6	1	5		2	4		1	5		1	5		2	4		2	4	
> 66	1	1	0		1	0		0	1		0	1		0	1		0	1	
Lobular inflammation (foci/field)^a^																			
< 2	17	2	15	0.055	3	14	0.548	4	13	1.000	5	12	0.290	4	13	1.000	3	14	0.548
2–4	5	3	2		2	3		1	4		0	5		1	4		2	3	
Ballooning^a^																			
Few	19	3	16	0.117	3	16	0.117	5	14	1.000	5	14	1.000	4	15	1.000	4	15	1.000
Many	3	2	1		2	1		0	3		0	3		1	2		1	2	
Nonalcoholic fatty liver disease activity score^a^																			
3–5	20	3	17	*0.043*	3	17	*0.043*	5	15	1.000	5	15	1.000	4	16	0.411	4	16	0.411
6–7	2	2	0		2	0		0	2		0	2		1	1		1	1	
Necroinflammatory grade^b^																			
Mild to moderate	20	3	17	*0.043*	3	17	*0.043*	5	15	1.000	5	15	1.000	4	16	0.411	4	16	0.411
Severe	2	2	0		2	0		0	2		0	2		1	1		1	1	
Brunt stage^c^																			
1	5	0	5	0.630	1	4	1.000	1	4	0.886	1	4	0.886	1	4	0.368	1	4	0.886
2	10	3	7		3	7		2	8		2	8		1	9		2	8	
3	5	2	3		1	4		1	4		1	4		2	3		2	3	
4	2	0	2		0	2		1	1		1	1		1	1		0	2	
Clinicopathological characteristics of HCCs																			
Differentiation^d^																			
Well	4	0	4	0.783	0	4	0.783	3	1	*0.029*	0	4	0.108	0	4	0.108	1	3	1.000
Moderately	13	4	9		4	9		2	11		2	11		2	11		3	10	
Poorly	5	1	4		1	4		0	5		3	2		3	2		1	4	
Portal vein invasion																			
Negative	10	1	9	0.323	1	9	0.323	3	7	0.624	3	7	0.624	3	7	0.624	2	8	1.000
Positive	12	4	8		4	8		2	10		2	10		2	10		3	9	
Pathological tumor-node-metastasis stage^e^																			
IA–IB	8	1	7	0.613	0	8	0.115	3	5	0.309	2	6	1.000	2	6	1.000	2	6	1.000
II–IIIB	14	4	10		5	9		2	12		3	11		3	11		3	11	

^a^According to Kleiner et al. (2005)

^b^According to the Brunt classification (1999)

^c^Used for assessment of hepatic fibrosis (Brunt et al. 1999)

^d^In accordance with the World Health Organization classification (Hirohashi et al. 2000)

^e^In accordance with the Union for International Cancer Control (UICC) classification (Brierley et al. 2017). *P* values of < 0.05 are in italics

On the other hand, lower expression of the *TRIM4* gene was significantly correlated with poorer tumor differentiation (*P* = 0.029) (Table 4). We then compared the levels of mRNA expression for the *TRIM4* gene in well, moderately and poorly differentiated HCCs with those of C samples (Supplementary Figure S2). Interestingly, *TRIM4* expression was initially increased during the establishment of cancer, and thereafter decreased during the process of dedifferentiation from well to poorly differentiated HCC. However, the small number of samples in each group in Tables 3 and 4 and Supplementary Figure S2 is a limitation, even though statistically significant differences were revealed among the groups.

## Discussion

It is well known that DNA methylation plays a key role in transcription regulation (Jones and Liang 2009). In addition, once DNA methylation status is altered during multistage carcinogenesis, such alteration is stably maintained through the maintenance mechanism mediated by DNA methyltransferase DNMT1 (Jones and Liang 2009). This suggests that genome-wide DNA methylation profiling of cancerous tissue would be a powerful tool for identifying genes whose expression levels are stably altered and which participate in carcinogenesis. Since we and another group have revealed that DNA methylation alterations actually occur even at the precancerous NASH stage (de Mello et al. 2017; Kuramoto et al. 2017), to identify truly “aberrant” DNA methylation in NASH-related HCCs, DNA methylation profiles of T samples were compared with those of C samples, and not with N samples. In this way, we identified aberrant DNA methylation at 19,281 CpG sites in NASH-related HCCs.

To identify DNA methylation alterations possibly resulting in alterations of expression, we next focused on Infinium probe CpG sites located near TSS and in CpG islands and their shores and shelves, which play an important role in expression regulation (Jones 2012). Four out of 20 signaling pathways including the top 3 pathways, in which possible alterations of expression occurred due to changes in DNA methylation, were related to NF-ΚB (Table 1). Since inflammatory cytokines can disturb the DNA methylation status of specific genes (Rokavec et al. 2016), it is feasible that expression alterations due to changes in the DNA methylation of NF-ΚB-related genes occur during the inflammatory process of NASH, thus promoting NF-KB signaling-related cell proliferation, tumorigenesis and epithelial–mesenchymal transition (EMT) (Kaltschmidt et al. 2019). In addition, Table 1 suggests that participation of the Wnt signaling pathway (Wands and Kim 2014) in NASH-related carcinogenesis may be at least partly attributable to expression alterations resulting from changes in DNA methylation. To further reveal the significance of these signaling pathways, mRNA expression of the representative genes, i.e., *PSMD6* (Islam et al. 2018), *TNFRSF10A* (Santonocito et al. 2013; Etemadi et al. 2015), *VANGLE2* (Acedo et al. 2019) and *LRRK1* (Lv et al. 2018), which were involved in the NF-ΚB signaling and/or Wnt signaling pathways shown in Table 1, was examined by real-time quantitative RT-PCR analysis. Significantly reduced mRNA expression of the four examined genes was observed in T samples relative to C samples (Supplementary Figure S3), further highlighting the significance of the NF-ΚB and Wnt signaling pathways during NASH-related hepatocarcinogenesis.

In addition to silencing of tumor-suppressor genes by DNA hypermethylation, overexpression of oncogenic genes due to DNA hypomethylation would play a potentially important role in tumorigenesis (Good et al. 2018). Considering that genes overexpressed in tumors may become potential therapeutic targets, we focused especially on genes that might become overexpressed by DNA hypomethylation. We pre-selected candidate genes that would potentially show an inverse correlation between DNA methylation and mRNA expression using TCGA multi-organ cancer data. This strategy may be promising to find candidate genes among a broad spectrum of cancers, but would potentially create a bias in revealing significant alterations during hepatocarcinogenesis: genes regulated by DNA methylation only in organs other than the liver and/or HCC may be included in the 24 examined genes. This may be the reason why a significant inverse correlation was revealed by our real-time quantitative RT-PCR analysis in only 6 of the 24 examined genes.

With regard to *DCAF4L2*, *CKLF*,* TRIM4*,* PRC1*,* UBE2C* and *TUBA1B,* we were able to confirm DNA hypomethylation, overexpression and an inverse correlation between DNA methylation and mRNA expression using our own C, N and T samples (*n* = 75) (Table 2). When we examined the correlation between DNA methylation level and mRNA expression in each of the C, N and T samples separately, inverse correlations of *DCAF4L2* and *TRIM4* were confirmed even in T samples alone, but were not revealed in N samples alone (Supplementary Table S5). This may be attributable to the decrease in the number of samples of each of C, N and T, compared to Table 2 which joined all 75 samples. Moreover, it may be difficult to demonstrate an inverse correlation unless analyzed with control C samples.

Even though significant alterations of mRNA expression from C to N and then to T samples were observed for the *CKLF*,* TRIM4*,* PRC1*,* UBE2C* and *TUBA1B* genes based on the Jonckheere–Terpstra trend test, alterations in N samples relative to C samples were more obvious in DNA methylation (Fig. 2) than in mRNA expression (Supplementary Figure S1), indicating that DNA methylation alterations precede and then may induce mRNA expression alterations in the precancerous N stages.

Although DNA hypomethylation and overexpression of *TRIM4* have been reported in neural tube defects (Zhang et al. 2019), no previous studies have yet revealed regulation of expression by DNA methylation of the *DCAF4L2*,* CKLF*,* TRIM4*,* PRC1*,* UBE2C* and *TUBA1B* genes. However, in addition to the TCGA data, the inverse correlation between DNA methylation and mRNA expression levels in our own cohort suggests the possibility of epigenetic regulation of these genes, although further confirmation using demethylating agents in vitro will be necessary.

Although overexpression of *DCAF4L2* has been observed in human colonic cancers (Wang et al. 2016), its association with HCCs regardless of their etiology (viral hepatitis, heavy alcohol consumption or NASH) has never been reported. Our data clearly revealed that overexpression of *DCAF4L2* is a common feature of NASH-related HCCs and viral hepatitis-related HCCs (Fig. 3). Since both DNA hypomethylation and overexpression of *DCAF4L2* were significantly correlated with clinicopathological parameters of N samples reflecting the severity of inflammation (NAS score and necroinflammatory grade), it appears that *DCAF4L2* abnormalities can be induced by the inflammatory process during the precancerous NASH stage. DNA hypomethylation occurring at the precancerous NASH stage (N samples) was clearly inherited by HCCs (T samples) (Fig. 2), suggesting that precancerous hepatocytes showing *DCAF4L2* overexpression might gain a growth advantage and show clonal expansion. DCAF4L2 is a member of the E3 ligase complex and reportedly mediates degradation of PPM1B, which negatively regulates NF-ΚB signaling (Angers et al. 2006). Introduction of *DCAF4L2* in cancer cell lines has been shown to promote EMT, and this is considered attributable to activation of NF-ΚB signaling (Wang et al. 2016). *DCAF4L2* overexpression and other abnormalities of NF-ΚB signaling shown in Table 1 may be mutually complementary for development and progression of NASH-related HCCs, and DCAF4L2 may therefore be a candidate therapeutic target in HCCs.

In non-cancerous liver tissue, *CKLF* overexpression was significantly correlated with clinicopathological parameters reflecting the severity of inflammation (Table 4). Since CKLF is a chemokine (Liu et al. 2018), we cannot completely rule out the possibility that CKLF is secreted by inflammatory cells. On the other hand, DNA hypomethylation was clearly strengthened in T samples, which generally contain fewer inflammatory cells than N samples (Fig. 2). It has been previously reported that malignant cells, such as ovarian cancer cells, secrete CKLF and thus promote the proliferation of cancer cells in an autocrine or paracrine manner (Liu et al. 2018), suggesting that HCC cells themselves might also overexpress *CKLF*. Furthermore, the levels of *CKLF* gene DNA methylation in NASH-related HCCs were the same as those in viral hepatitis-related HCCs (Fig. 3), indicating that CKLF could be a potential target in HCCs regardless of etiology. In addition, although association of *CKLF* abnormalities with HCCs has not been reported, a high level of *CKLF* expression in HCC tissues was just reported at the time when this manuscript was in the process of preparation (Zhu et al. 2019). CKLF is a functional ligand for CCR4 (CC chemokine receptor 4) and reportedly activates the NF-ΚB signaling pathway in lung epithelial cells (Li et al. 2014). This analogous role in such epithelial cells again highlights the significance of the NF-ΚB signaling pathway.

Tripartite motif (TRIM*)* family proteins, most of which have E3 ubiquitin ligase activity, have various functions in cellular processes such as apoptosis and autophagy, and show both oncogenic and tumor-suppressive activities (Hatakeyama 2017). With regard to TRIM4, recent studies have revealed that it induces mitochondrial aggregation, increases the level of mitochondrial reactive oxygen species in the presence of H_2_O_2_ (Tomar et al. 2015) and participates in antiviral innate immunity (Yan et al. 2014). Although an association of TRIM4 with carcinogenesis has not been clarified in any organ, our data clearly revealed that NASH-related HCC-specific DNA hypomethylation (Fig. 3), which occurred even at the precancerous NASH stage (in N samples) and was further strengthened in T samples (Fig. 2), is associated with overexpression of TRIM4. Interestingly, as shown in Supplementary Figure S2, TRIM4 appears to be important only in the transitional stage from C to T, but not during further progression to poorly differentiated HCC. Taken together, the data suggest that the significance of TRIM4 in NASH-related carcinogenesis may be worth closer scrutiny.

A microtubule-associated protein, PRC1, helps to sequester the β-catenin destruction complex consisting of Axin, APC, GSK3 and CK1 at the microtubule, stabilizing the cytoplasmic β-catenin and acting as a Wnt signaling activator (Chen et al. 2017). Even though epigenetic regulation of *PRC1* expression in human cancers has not been reported, overexpression of *PRC1* has been described in many cancers, such as urothelial carcinoma of the urinary bladder (Su et al. 2019) and adenocarcinoma of the colon (Subramaniyan et al. 2016). In HCCs, enhanced expression of *PRC1* is reportedly associated with early recurrence and poorer prognosis in patients with HCCs (Chen et al. 2016). These findings appear to be consistent with the overexpression we observed in the present samples of moderately to poorly differentiated HCCs (Table 4).

UBE2C is another integral component of the ubiquitin proteasome and partners the anaphase-promoting complex (Jin et al. 2019). The oncogenic significance of UBE2C has been clearly demonstrated in *UBE2C-*transgenic mice, which are prone to develop both spontaneous and carcinogen-induced tumors with chromosome aneuploidy (Jin et al. 2019). *UBE2C* overexpression has already been reported in many cancer types, such as lung (Jin et al. 2019) and kidney (Wei et al. 2019) cancers. *UBE2C* overexpression in moderately to poorly differentiated HCCs in the present study (Table 4) was consistent with previous studies demonstrating a correlation with clinicopathological aggressiveness and poorer prognosis of HCCs (Xiong et al. 2019; Ieta et al. 2007). Although the NASH-related etiology of HCCs showing overexpression of *UBE2C* has never been addressed in previous reports, the present study clearly demonstrated *UBE2C* DNA hypomethylation in both precancerous NASH and NASH-related HCCs (Fig. 2).

Dynamic α–β tubulin heterodimerization modulates microtubule remodeling, possibly influencing cytoskeletal rearrangement, cell migration and motility, as well as the cell cycle in cancers (Hohmann and Dehghani 2019). Although the relationship of TUBA1B to human cancers has been poorly documented, one previous immunohistochemical study has demonstrated that higher expression of TUBA1B was a significant predictor of overall survival in HCC patients (Lu et al. 2013). In addition, TUBA1B knockout in HCC cell lines was shown to inhibit cell proliferation and increase sensitivity to the antimicrotubule agent, paclitaxel (Lu et al. 2013). Although this previous study did not address the etiology of HCCs with TUBA1B overexpression, the present study clearly revealed that alterations of *TUBA1B* gene DNA methylation had already occurred at the precancerous NASH stage (Fig. 2) and in a NASH-specific manner (Fig. 3). Whether or not the level of TUBA1B expression can become a predictor of sensitivity to antimicrotubule agents is an issue for further investigation in NASH-related HCCs.

In this study, genome-wide DNA methylation analysis using tissue samples of HCCs that were clearly derived from the precancerous background of NASH identified important tumor-related genes showing DNA hypomethylation and overexpression, such as *DCAF4L2*, *CKLF*, *TRIM4*, *PRC1*, *UBE2C* and *TUBA1B*. Genome-wide DNA methylation analysis using human clinical samples is a powerful tool for identifying tumor-related genes with significant expression alterations. Although it remains to be investigated whether these gene products can become therapeutic targets for NASH-related HCCs, it is clear that DNA methylation alterations occurring under the necroinflammatory conditions of NASH are inherited by or strengthened in NASH-related HCCs and participate in NASH-related hepatocarcinogenesis through aberrant expression of tumor-related genes.

## Electronic supplementary material

Below is the link to the electronic supplementary material.

Supplementary file1 (PDF 950 kb)

## Abbreviations

C: Normal control liver tissue
GO: Gene ontology
HBV: Hepatitis B virus
HCC: Hepatocellular carcinoma
HCV: Hepatitis C virus
EMT: Epithelial mesenchymal transition
NAS: Non-alcoholic fatty liver disease activity score
NASH: Non-alcoholic steatohepatitis
N: Non-cancerous liver tissue obtained from patients with NASH-related HCCs
RT: Reverse transcription
TNM: Tumor–node–metastasis
