# Supplementary material for: Aberrant DNA methylation results in altered gene expression in non-alcoholic steatohepatitis-related hepatocellular carcinomas
Source: J Cancer Res Clin Oncol. 2020 Jul 19;146(10):2461–77. doi: 10.1007/s00432-020-03298-4 (PMC7467955; doi:10.1007/s00432-020-03298-4)
Supplement: Supplementary file 1 — Supplementary file1 (PDF 950 kb) [file 432_2020_3298_MOESM1_ESM.pdf]

Supplementary Table S1 Clinicopathological characteristics of patients with hepatocellular carcinoma (HCC)

(A) Non-alcoholic steatohepatitis-related HCCs

Clinicopathological parameters		Number of patients (n=22)
Age (years)		71.2 ± 7.65
Sex	Male	20
	Female	2
Histological findings in non-cancerous liver tissue		
Steatosis (%)[a]	5-33	15
	33-66	6
	> 66	1
Lobular inflammation (foci/field)[a]	< 2	17
	2-4	5
Ballooning[a]	Few	19
	Many	3
Nonalcoholic fatty liver disease activity score[a]	3	13
	4	5
	5	2
	6	1
	7	1
Necroinflammatory grading[b]	Mild	16
	Moderate	4
	Severe	2
Brunt stage[c]	1	5
	2	10
	3	5
	4	2
Characteristics of HCCs		
Differentiation[d]	Well	4
	Moderately	13
	Poorly	5
Portal vein invasion	Negative	10
	Positive	12
Pathological Tumor-Node-Metastasis stage[e]	IA	1
	IB	7
	II	12
	IIIA	1
	IIIB	1

(B) Viral hepatitis-related HCCs

Clinicopathological parameters		Number of patients (n=36)
Age (years)		60.4 ± 10.6
Sex	Male	31
	Female	5
Hepatitis virus	B	13
	C	22
	B and C	1
Characteristics of HCCs		
Differentiation[d]	Well	8
	Moderately	16
	Poorly	12
Portal vein invasion	Negative	16
	Positive	20
Pathological Tumor-Node-Metastasis stage[e]	IA	0
	IB	11
	II	24
	IIIA	1
	IIIB	0

[a]According to Kleiner et al (2005). [b]According to the Brunt classification (1999). [c]Used for assessment of hepatic fibrosis (Brunt et al. 1999). [d]In accordance with the World Health Organization classification (Hirohashi et al. 2000). [e]In accordance with the Union for International Cancer Control (UICC) classification (Brierley et al. 2017).

Supplementary Table S2 List of primers used for quantitative reverse transcription-PCR reaction

Gene	Primer	Sequence
GAPDH	Forward	5`tgtgggcaaggtcatccct3`
	Reverse	5`gccctccgacgcctgcttca3`
CKLF	Forward	5`gtgtttgcacttgtgacag3`
	Reverse	5`gattgaacagaagcttccg3`
DCAF4L2	Forward	5`aaccctctacgtccctaat3`
	Reverse	5`tgattcagtgaggcccagca3`
PRC1	Forward	5`gaggactacacagaaagtct3`
	Reverse	5`tcaaactctaagaaaagcct3`
TRIM4	Forward	5`cagaatccaaaagaagtgtt3`
	Reverse	5`cagctaggtttacagccact3`
TUBA1B	Forward	5`ccgctacttaagaggctcca3`
	Reverse	5`catagtggctagggattagga3`
PSMD6	Forward	5`cctctgccggataggtgacaaag3`
	Reverse	5`atatccaatcggtgacccagg3`
TNFRSF10A	Forward	5`gtccaccaggatctcatagatcag3`
	Reverse	5`ggaagcattggtgtaacccacac3`
VANGLE2	Forward	5`gggatgacaactgggggaaacg3`
	Reverse	5`gtgaggtcatcatgggagatgctg3`
LRRK1	Forward	5`acatgagatttcctgcaagagcctg3`
	Reverse	5`tggaaaatcagctgtcgcagc3`
UBE2C	Forward	5`gctgcccagcctgtccttgt3`
	Reverse	5`caaaacaaaaataccacagctca3`
CD44	Forward	5`gatttgaatataacctgccgct3`
	Reverse	5`ctattgaaagccttgcagaggt3`
HDAC11	Forward	5`atcgtgaagcgggatgagct3`
	Reverse	5`gtgcgcttctggtacccgcc3`
INPP5D	Forward	5`ggcgagggctgcattgcc3`
	Reverse	5`agatgggcagctgcgtttctgtg3`
MAPK13	Forward	5`acctggtgatgcccttcat3`
	Reverse	5`accaggtactggatcttct3`
PMEPA1	Forward	5`gaaggatgcctgtggccct3`
	Reverse	5`gaggcggggcgtagacct3`
PRAME	Forward	5`gcacgtgatgaaccccttgga3`
	Reverse	5`atgcatcacatccccttccga3`
RIPK3	Forward	5`ccaaccgaaccatcactcgt3`
	Reverse	5`gcagctcagccaatgaagg3`
RPS6KA1	Forward	5`ggctccggccctgatggggca3`
	Reverse	5`gtatagcttattccagtcaa3`
SH3BP1	Forward	5`gaccaggcccagctggatgca3`
	Reverse	5`tggatcagcgcctcgacgac3`
SORD	Forward	5`gcttcctgacaatgtcacct3`
	Reverse	5`ggcatggatccccacagaaa3`
TESC	Forward	5`ctgttccacatgtacgact3`
	Reverse	5`cgatattcttccagagtgat3`
VIM	Forward	5`tcagacaggatgttgacaat3`
	Reverse	5`caaagattccactttgcgtt3`
ABCC3	Forward	5`ctcctggtggggcttcctggt3`
	Reverse	5`gatcagcgactgcatcatgga3`
CLDN4	Forward	5`cctggagactgatcccctct3`
	Reverse	5`accctcccaggctcattagt3`
KRT19	Forward	5`gaaagctgccttggaagaca3`
	Reverse	5`atatgcgccagctgggctcca3`
FZD6	Forward	5`cattttcttcctctcgcaaatc3`
	Reverse	5`tttgcagaggaaagtttcaatg3`
CARD14	Forward	5`cggcagaccttccgcagc3`
	Reverse	5`ctccgagacatcaagccttcc3`
CHMP4C	Forward	5`agaagccctggagaactcac3`
	Reverse	5`tatcctgttgctctgtgatctc3`

Supplementary Table S3 The 681 probes for which call proportions in all examined tissue samples were less than 90%

TargetID[a]	Chromosome	Gene symbol	Call proportion (%)[b]
cg00149061	4	*UGT2A3*	88.8
cg00211215	6	*HLA-DRB1*	88.8
cg00409917	6	*HCG4B*	88.8
cg00639218	Y	*NA*	88.8
cg00843105	19	*ZNF234*	88.8
cg01336390	6	*NA*	88.8
cg01479912	2	*PDIA6*	88.8
cg01519225	6	*NA*	88.8
cg01757887	Y	*TTTY4C*	88.8
cg02107461	Y	*NA*	88.8
cg02606988	Y	*TSPY4*	88.8
cg02775404	22	*NA*	88.8
cg03035657	16	*NA*	88.8
cg03344051	6	*HLA-DQB1*	88.8
cg03398989	6	*NA*	88.8
cg03478313	15	*NA*	88.8
cg03673787	17	*GAA*	88.8
cg04016144	Y	*TSPY4*	88.8
cg04691144	Y	*NLGN4Y*	88.8
cg04840108	4	*TLR6*	88.8
cg05176970	17	*NXN*	88.8
cg05372444	7	*UPK3B*	88.8
cg05751189	2	*SLC9A4*	88.8
cg06350542	13	*MCF2L*	88.8
cg06407043	12	*NA*	88.8
cg06576965	Y	*TTTY18*	88.8
cg08357313	Y	*RBMY1A3P*	88.8
cg08634464	19	*ZNF57*	88.8
cg08703522	17	*NA*	88.8
cg09081202	Y	*NA*	88.8
cg09460641	Y	*NA*	88.8
cg09616536	3	*ADAMTS9-AS2*	88.8
cg10182287	14	*ATXN3*	88.8
cg10799208	Y	*TTTY10*	88.8
cg10811597	Y	*TTTY14*	88.8
cg11256764	5	*NA*	88.8
cg11844537	10	*TCERG1L*	88.8
cg11888470	6	*HLA-DRB6*	88.8
cg12017057	20	*TRIB3*	88.8
cg13017022	5	*ZMAT2*	88.8
cg14273923	Y	*NA*	88.8
cg14323910	6	*HLA-DQB1*	88.8
cg14926231	2	*NA*	88.8
cg15070894	6	*HCG4B*	88.8
cg15080433	2	*LY75-CD302*	88.8
cg15422579	Y	*EIF1AY*	88.8
cg16003256	1	*NA*	88.8
cg16223220	6	*HLA-H*	88.8
cg16248432	12	*DIP2B*	88.8

cg16535653	6	*NA*	88.8
cg17089062	4	*NA*	88.8
cg17972491	Y	*AMELY*	88.8
cg17982178	6	*NA*	88.8
cg18825597	8	*NA*	88.8
cg18827503	13	*NA*	88.8
cg21370869	6	*SMIM8*	88.8
cg21685655	7	*PON2*	88.8
cg21692241	6	*HLA-DQA1*	88.8
cg22025232	6	*HCG4B*	88.8
cg23130832	10	*TCERG1L*	88.8
cg23316161	12	*ZNF605*	88.8
cg24680646	13	*TRPC4*	88.8
cg24751894	6	*HCG4B*	88.8
cg24973221	10	*INPP5A*	88.8
cg25601879	10	*NA*	88.8
cg25914522	Y	*NA*	88.8
cg26046487	Y	*HSFY1*	88.8
cg26198148	Y	*TMSB4Y*	88.8
cg26294145	14	*NA*	88.8
cg26482585	6	*NA*	88.8
cg26983535	Y	*EIF1AY*	88.8
cg27128560	10	*COL17A1*	88.8
cg27433982	Y	*ZFY*	88.8
cg27509967	Y	*KDM5D*	88.8
cg27575622	17	*RPH3AL*	88.8
cg00063477	Y	*EIF1AY*	87.5
cg00113675	6	*HLA-H*	87.5
cg00169792	20	*SALL4*	87.5
cg00213748	Y	*NA*	87.5
cg00214611	Y	*TMSB4Y*	87.5
cg00271873	Y	*NA*	87.5
cg00648423	6	*NA*	87.5
cg00695324	X	*TEX13B*	87.5
cg00876332	Y	*BCORP1*	87.5
cg01426558	Y	*DDX3Y*	87.5
cg01499815	6	*NA*	87.5
cg01717482	3	*NA*	87.5
cg01900066	Y	*EIF1AY*	87.5
cg02011394	Y	*TSPY8*	87.5
cg02802508	Y	*TSPY4*	87.5
cg02914047	X	*RNF128*	87.5
cg03231275	6	*BTNL2*	87.5
cg03441493	Y	*NA*	87.5
cg03830006	17	*ENPP7*	87.5
cg04351468	Y	*RBMY1A3P*	87.5
cg04461745	17	*NA*	87.5
cg04790916	Y	*UTY*	87.5
cg04958669	Y	*TSPY4*	87.5
cg05276972	11	*NA*	87.5
cg05672930	Y	*TXLNG2P*	87.5
cg06237805	Y	*NA*	87.5

cg06587249	19	*ZNF71*	87.5
cg06924902	8	*ZFAT-AS1*	87.5
cg07075387	4	*NA*	87.5
cg07368937	5	*IRX4*	87.5
cg08268047	1	*FMO2*	87.5
cg08596608	Y	*GYG2P1*	87.5
cg09052185	6	*HLA-DQA1*	87.5
cg09312897	6	*HLA-DQB1*	87.5
cg09728865	Y	*TBL1Y*	87.5
cg09804407	Y	*NLGN4Y*	87.5
cg09829904	Y	*ZFY*	87.5
cg10051237	Y	*NA*	87.5
cg10115321	6	*CSNK2B*	87.5
cg10348826	3	*NA*	87.5
cg10620659	Y	*EIF1AY*	87.5
cg10666546	Y	*NA*	87.5
cg10691859	Y	*NCRNA00185*	87.5
cg10804687	6	*NA*	87.5
cg11896151	7	*FAM20C*	87.5
cg12833683	8	*VPS13B*	87.5
cg13117582	17	*NA*	87.5
cg13654344	Y	*SRY*	87.5
cg14029254	Y	*RPS4Y2*	87.5
cg14449180	6	*HCG4B*	87.5
cg14492024	Y	*TXLNG2P*	87.5
cg14629010	X	*ZNF157*	87.5
cg15161973	10	*TCERG1L*	87.5
cg15197499	Y	*TBL1Y*	87.5
cg15214366	13	*CDX2*	87.5
cg15408512	10	*TUBGCP2*	87.5
cg15810474	Y	*TSPY1*	87.5
cg15853771	2	*NA*	87.5
cg16443079	5	*NA*	87.5
cg16558846	14	*NA*	87.5
cg16626452	Y	*TSPY4*	87.5
cg17107246	1	*SLC25A24*	87.5
cg17480035	6	*HLA-DRB1*	87.5
cg18085787	Y	*NA*	87.5
cg18423635	6	*NA*	87.5
cg18697348	3	*SELK*	87.5
cg19043679	5	*RGS14*	87.5
cg19244032	Y	*NLGN4Y*	87.5
cg19432688	2	*NA*	87.5
cg19676100	2	*NA*	87.5
cg19776366	17	*BAIAP2*	87.5
cg20256738	Y	*TSPY2*	87.5
cg20694666	13	*N6AMT2*	87.5
cg21810411	6	*HLA-DRB1*	87.5
cg22047338	19	*C19orf35*	87.5
cg22134413	5	*NA*	87.5
cg22805356	15	*NA*	87.5
cg23472400	7	*TAF6*	87.5

cg24845318	X	*FRMPD4*	87.5
cg25644740	6	*HCG4B*	87.5
cg25918849	Y	*TTTY13*	87.5
cg27425262	2	*PSD4*	87.5
cg27443332	Y	*NLGN4Y*	87.5
cg27521160	19	*PSG4*	87.5
cg27578568	Y	*TTTY19*	87.5
cg27636129	Y	*SRY*	87.5
cg00212031	Y	*TTTY14*	86.3
cg00567916	9	*NTNG2*	86.3
cg00903245	Y	*FAM197Y5*	86.3
cg01061520	Y	*NA*	86.3
cg01141334	Y	*NA*	86.3
cg01215343	Y	*NA*	86.3
cg01238044	22	*GSTT1*	86.3
cg01493009	13	*FOXO1*	86.3
cg01644972	Y	*EIF1AY*	86.3
cg01647917	19	*GZMM*	86.3
cg01988452	Y	*EIF1AY*	86.3
cg02056550	Y	*TTTY18*	86.3
cg02126249	Y	*TSPY4*	86.3
cg02233190	Y	*EIF1AY*	86.3
cg02577797	Y	*ZFY*	86.3
cg02730008	Y	*TMSB4Y*	86.3
cg03155755	Y	*NA*	86.3
cg03443143	Y	*FAM197Y2*	86.3
cg03535417	Y	*FAM197Y2*	86.3
cg03601053	Y	*DDX3Y*	86.3
cg03767353	Y	*NA*	86.3
cg04448376	Y	*UTY*	86.3
cg04817258	Y	*NA*	86.3
cg04831594	Y	*NA*	86.3
cg05202434	Y	*TTTY19*	86.3
cg05362314	4	*NA*	86.3
cg05865243	Y	*TSPY2*	86.3
cg05940236	Y	*DDX3Y*	86.3
cg06064954	6	*NA*	86.3
cg06502680	Y	*TXLNG2P*	86.3
cg06636270	Y	*TTTY20*	86.3
cg06844719	16	*NA*	86.3
cg06850186	7	*NA*	86.3
cg08147391	X	*CXorf56*	86.3
cg08242338	Y	*TSPY2*	86.3
cg08820785	Y	*EIF1AY*	86.3
cg09194930	16	*MT1L*	86.3
cg09228985	Y	*BCORP1*	86.3
cg09230658	Y	*NA*	86.3
cg09732580	Y	*NA*	86.3
cg10018004	6	*HLA-A*	86.3
cg11507597	12	*NA*	86.3
cg11942206	19	*PSG1*	86.3
cg11967332	1	*SLC25A24*	86.3

cg13141283	13	*PCDH20*	86.3
cg13618458	Y	*NA*	86.3
cg13808036	Y	*RBMY2FP*	86.3
cg14442616	Y	*DDX3Y*	86.3
cg14463736	Y	*TMSB4Y*	86.3
cg15329860	Y	*KDM5D*	86.3
cg15591384	6	*HLA-DRB6*	86.3
cg16662938	10	*GUCY2GP*	86.3
cg16886051	11	*GVINP1*	86.3
cg17025908	1	*NA*	86.3
cg17370981	13	*ATP4B*	86.3
cg17430262	Y	*NA*	86.3
cg17560699	Y	*UTY*	86.3
cg17834650	Y	*FAM197Y5*	86.3
cg18168924	Y	*PRKY*	86.3
cg18442075	17	*NKIRAS2*	86.3
cg19600472	6	*HCG4B*	86.3
cg20401549	Y	*PRKY*	86.3
cg20764275	Y	*NA*	86.3
cg21234342	6	*GPR116*	86.3
cg21543103	5	*PAPD7*	86.3
cg22627029	6	*HLA-DRB6*	86.3
cg23340935	2	*DIRC3*	86.3
cg23877661	X	*GABRQ*	86.3
cg25443613	Y	*RPS4Y1*	86.3
cg25538674	Y	*TSPY8*	86.3
cg25613667	5	*WDR55*	86.3
cg25730351	11	*ANO1*	86.3
cg25815185	Y	*KDM5D*	86.3
cg26488634	Y	*NA*	86.3
cg26651280	16	*GALNS*	86.3
cg26924012	15	*SPATA5L1*	86.3
cg26928789	Y	*TTTY10*	86.3
cg27214488	Y	*NLGN4Y*	86.3
cg27325772	Y	*NA*	86.3
cg00050873	Y	*FAM197Y5*	85.0
cg00308367	Y	*KDM5D*	85.0
cg00543493	Y	*TSPY4*	85.0
cg00599377	Y	*NA*	85.0
cg00679624	Y	*NA*	85.0
cg00727777	8	*NA*	85.0
cg00762184	Y	*TTTY15*	85.0
cg00988056	14	*NA*	85.0
cg01073572	Y	*NLGN4Y*	85.0
cg01232331	2	*NA*	85.0
cg01523029	Y	*TSPY4*	85.0
cg01943289	Y	*RPS4Y2*	85.0
cg01984154	Y	*RPS4Y1*	85.0
cg02105393	Y	*ZFY*	85.0
cg02522936	Y	*TTTY10*	85.0
cg02839557	Y	*TBL1Y*	85.0
cg03052502	Y	*FAM197Y2*	85.0

cg03266527	Y	*UTY*	85.0
cg03416979	Y	*NA*	85.0
cg03430010	Y	*UTY*	85.0
cg03533500	Y	*NA*	85.0
cg03905640	Y	*FAM41AY2*	85.0
cg04042030	Y	*TBL1Y*	85.0
cg04387098	X	*EBP*	85.0
cg04419680	Y	*NLGN4Y*	85.0
cg04576441	Y	*NA*	85.0
cg04840163	Y	*ZFY*	85.0
cg05098815	Y	*RBMY3AP*	85.0
cg05128824	Y	*DDX3Y*	85.0
cg05367916	Y	*NA*	85.0
cg05408674	Y	*NA*	85.0
cg05480730	Y	*PRKY*	85.0
cg05621349	Y	*TXLNG2P*	85.0
cg05954446	Y	*AMELY*	85.0
cg06008419	8	*NA*	85.0
cg06060201	Y	*FAM224A*	85.0
cg06075393	20	*RASSF2*	85.0
cg06231362	Y	*NA*	85.0
cg06461588	20	*DNAJC5*	85.0
cg06634367	17	*RCVRN*	85.0
cg06647930	22	*PRR5-ARHGAP8*	85.0
cg07559763	8	*NA*	85.0
cg07939587	Y	*NA*	85.0
cg07950979	Y	*UTY*	85.0
cg08053115	Y	*TTTY1*	85.0
cg08160949	Y	*NA*	85.0
cg08314875	Y	*DDX3Y*	85.0
cg08593141	Y	*NA*	85.0
cg08816194	Y	*NLGN4Y*	85.0
cg09408193	Y	*NA*	85.0
cg09595415	Y	*SRY*	85.0
cg09856092	Y	*DDX3Y*	85.0
cg10239257	Y	*TSPY3*	85.0
cg10267609	Y	*RBMY2FP*	85.0
cg10363397	Y	*NA*	85.0
cg10536901	1	*SLC25A24*	85.0
cg10557773	Y	*ZFY*	85.0
cg10646950	Y	*TTTY9A*	85.0
cg10772532	14	*CEP128*	85.0
cg11019305	5	*UBTD2*	85.0
cg11225091	Y	*EIF1AY*	85.0
cg11684211	Y	*TTTY14*	85.0
cg11816202	Y	*TTTY14*	85.0
cg11919336	12	*HCAR2*	85.0
cg13268984	Y	*NA*	85.0
cg13419214	Y	*TTTY8B*	85.0
cg13636640	20	*DNMT3B*	85.0
cg13830619	12	*NA*	85.0
cg14104700	13	*MIPEPP3*	85.0

cg14151065	Y	*NA*	85.0
cg14669863	1	*HCN3*	85.0
cg14741114	Y	*TTTY15*	85.0
cg14742615	Y	*NA*	85.0
cg15059553	Y	*EIF1AY*	85.0
cg15281205	Y	*TTTY14*	85.0
cg15345074	Y	*TTTY14*	85.0
cg16292375	Y	*FAM224A*	85.0
cg16894943	Y	*FAM224A*	85.0
cg17520080	1	*LUZP1*	85.0
cg18015625	2	*B3GALT1*	85.0
cg18058072	Y	*ZFY*	85.0
cg18077436	Y	*DDX3Y*	85.0
cg18163559	Y	*NA*	85.0
cg20951645	X	*BGN*	85.0
cg21164095	1	*BAI2*	85.0
cg22696546	1	*CNST*	85.0
cg24120210	7	*RADIL*	85.0
cg24183504	Y	*NA*	85.0
cg24837623	Y	*ZFY*	85.0
cg25395304	19	*ZNF234*	85.0
cg25427172	Y	*NA*	85.0
cg25667057	Y	*NA*	85.0
cg25705492	Y	*TSPY4*	85.0
cg25756647	Y	*TXLNG2P*	85.0
cg25971649	1	*CD58*	85.0
cg26497631	Y	*NA*	85.0
cg26590106	6	*HLA-DRB1*	85.0
cg27441354	7	*NA*	85.0
cg00035864	Y	*TTTY18*	83.8
cg00061679	Y	*DAZ1*	83.8
cg00103771	6	*HLA-DRB6*	83.8
cg00121626	Y	*BCORP1*	83.8
cg00272582	Y	*ZFY*	83.8
cg00311963	Y	*TTTY23*	83.8
cg00479827	Y	*NCRNA00185*	83.8
cg00742920	13	*NA*	83.8
cg00789540	Y	*NA*	83.8
cg00886571	14	*NA*	83.8
cg00975375	Y	*NA*	83.8
cg01498999	Y	*NLGN4Y*	83.8
cg01828798	Y	*TTTY19*	83.8
cg02012379	Y	*RPS4Y2*	83.8
cg02050847	Y	*RPS4Y2*	83.8
cg02129146	Y	*NA*	83.8
cg02340092	Y	*NLGN4Y*	83.8
cg02352633	Y	*RBMY2EP*	83.8
cg02616328	Y	*ZFY*	83.8
cg02751273	2	*NA*	83.8
cg02764429	15	*PRKXP1*	83.8
cg02842889	Y	*ZFY*	83.8
cg02847425	7	*UPK3B*	83.8

cg03123709	Y	*TMSB4Y*	83.8
cg03672679	Y	*NA*	83.8
cg03683899	Y	*ZFY*	83.8
cg03750315	Y	*EIF1AY*	83.8
cg03769088	Y	*NA*	83.8
cg03827298	Y	*RBMY2FP*	83.8
cg04123665	Y	*NA*	83.8
cg04169747	Y	*SRY*	83.8
cg04193779	Y	*TTTY20*	83.8
cg04303809	Y	*NA*	83.8
cg04559508	Y	*NA*	83.8
cg04879348	2	*GCC2*	83.8
cg05378695	Y	*TTTY8B*	83.8
cg05530472	Y	*AMELY*	83.8
cg05544622	Y	*TSPY1*	83.8
cg05557315	Y	*NA*	83.8
cg05725925	Y	*TXLNG2P*	83.8
cg05890011	Y	*TTTY15*	83.8
cg05964935	Y	*NA*	83.8
cg06206057	6	*NA*	83.8
cg06322277	Y	*RPS4Y2*	83.8
cg06479204	Y	*UTY*	83.8
cg06587955	Y	*TSPY3*	83.8
cg06628792	Y	*TTTY14*	83.8
cg06865724	Y	*TTTY5*	83.8
cg07731488	Y	*TXLNG2P*	83.8
cg07747963	Y	*RPS4Y2*	83.8
cg08258654	Y	*TSPY4*	83.8
cg08528516	Y	*DDX3Y*	83.8
cg08589776	4	*NA*	83.8
cg08673225	Y	*RPS4Y1*	83.8
cg08702825	Y	*NA*	83.8
cg08739478	Y	*NA*	83.8
cg08921682	Y	*TBL1Y*	83.8
cg10067523	Y	*TTTY11*	83.8
cg10076560	Y	*TXLNG2P*	83.8
cg10172760	Y	*EIF1AY*	83.8
cg10338539	Y	*SRY*	83.8
cg10593480	Y	*EIF1AY*	83.8
cg10698069	Y	*DDX3Y*	83.8
cg11021362	Y	*TTTY13*	83.8
cg11131351	Y	*ZFY*	83.8
cg11193865	14	*DLK1*	83.8
cg11454325	10	*GPR123*	83.8
cg11898347	Y	*SRY*	83.8
cg12126656	10	*INPP5A*	83.8
cg13252613	Y	*TTTY23B*	83.8
cg13281118	X	*GAGE2C*	83.8
cg13308744	Y	*EIF1AY*	83.8
cg13311805	11	*OR4S2*	83.8
cg13749113	15	*NA*	83.8
cg13805219	Y	*NA*	83.8

cg13845521	Y	*TTTY14*	83.8
cg14005657	Y	*TSPY3*	83.8
cg14133106	Y	*NA*	83.8
cg14157445	Y	*TTTY15*	83.8
cg14526044	Y	*ZFY*	83.8
cg14671357	Y	*TTTY12*	83.8
cg14778208	Y	*TTTY16*	83.8
cg14931215	Y	*KDM5D*	83.8
cg14972466	Y	*ZFY*	83.8
cg15014361	6	*HLA-DQA1*	83.8
cg15141401	11	*OR4C11*	83.8
cg15273954	Y	*TTTY15*	83.8
cg15429127	Y	*DDX3Y*	83.8
cg15462332	Y	*RBMY1D*	83.8
cg15535896	18	*NA*	83.8
cg15563434	Y	*NA*	83.8
cg15662272	Y	*KDM5D*	83.8
cg15682993	Y	*TXLNG2P*	83.8
cg15700967	Y	*TBL1Y*	83.8
cg15746461	Y	*TTTY15*	83.8
cg15849038	Y	*NA*	83.8
cg16733676	1	*SLC25A24*	83.8
cg17422543	2	*NA*	83.8
cg17626671	7	*EPHA1-AS1*	83.8
cg17837162	Y	*TSPY4*	83.8
cg17939569	Y	*DAZ2*	83.8
cg18032798	Y	*TMSB4Y*	83.8
cg19726599	4	*AFAP1*	83.8
cg20864678	Y	*TXLNG2P*	83.8
cg21114725	3	*NA*	83.8
cg21442271	22	*NA*	83.8
cg22051787	Y	*RBMY1J*	83.8
cg24393100	Y	*NA*	83.8
cg25518695	Y	*NLGN4Y*	83.8
cg25598710	2	*NA*	83.8
cg25704368	Y	*TTTY20*	83.8
cg26517491	Y	*KDM5D*	83.8
cg27049643	Y	*KDM5D*	83.8
cg27112983	16	*NA*	83.8
cg27545697	Y	*NA*	83.8
cg27611726	Y	*TBL1Y*	83.8
cg00978846	6	*HLA-DQA1*	82.5
cg01550813	7	*NA*	82.5
cg02262167	13	*NA*	82.5
cg03187668	11	*OR4C6*	82.5
cg03466083	5	*NA*	82.5
cg03999583	3	*NA*	82.5
cg08231637	6	*HLA-DRB1*	82.5
cg09462281	19	*NCLN*	82.5
cg10325211	2	*NA*	82.5
cg10769535	1	*DNM3OS*	82.5
cg13685349	14	*STON2*	82.5

cg15225267	6	*NA*	82.5
cg17446339	11	*HTATIP2*	82.5
cg17901463	1	*GSTM1*	82.5
cg18433519	6	*KIAA0319*	82.5
cg18474443	10	*PFKP*	82.5
cg18792536	7	*UPK3B*	82.5
cg26657539	4	*NA*	82.5
cg27107292	6	*HLA-DRB1*	82.5
cg27257987	19	*PSG4*	82.5
cg01469864	5	*CPLX2*	81.3
cg01790920	5	*IRX1*	81.3
cg03271827	4	*PCGF3*	81.3
cg04057378	20	*NA*	81.3
cg07643074	4	*NA*	81.3
cg09988111	16	*NA*	81.3
cg10123347	14	*DDHD1*	81.3
cg10747483	19	*NA*	81.3
cg11727304	7	*GALNTL5*	81.3
cg12074150	2	*NA*	81.3
cg12391576	6	*HLA-DPA1*	81.3
cg13910785	6	*HLA-DRB1*	81.3
cg14181874	2	*NA*	81.3
cg14186264	6	*C6orf58*	81.3
cg21697769	2	*AGAP1*	81.3
cg22304543	8	*NA*	81.3
cg22802296	1	*PBX1*	81.3
cg01546248	22	*SMTN*	80.0
cg01600516	17	*ALOX12*	80.0
cg01914365	3	*NA*	80.0
cg02234672	13	*NA*	80.0
cg03183700	8	*ANK1*	80.0
cg06001854	5	*SCAMP1*	80.0
cg07765982	Y	*TTTY1*	80.0
cg07904290	16	*NA*	80.0
cg08299859	10	*NA*	80.0
cg08433110	6	*GMDS*	80.0
cg10217052	6	*HLA-DQA1*	80.0
cg12770425	6	*NA*	80.0
cg14555649	6	*DKFZP686I15217*	80.0
cg14803009	2	*MSH2*	80.0
cg14834300	1	*NA*	80.0
cg15954684	3	*NA*	80.0
cg22993154	6	*NA*	80.0
cg24760581	6	*HLA-DRB1*	80.0
cg01317752	X	*VCX*	78.8
cg03391002	12	*WSCD2*	78.8
cg05028071	X	*TFE3*	78.8
cg08335702	12	*MTERFD3*	78.8
cg11170468	11	*OR8G1*	78.8
cg14457452	9	*CDK5RAP2*	78.8
cg14799809	6	*HLA-DQA1*	78.8
cg15577010	2	*NMUR1*	78.8

cg16034991	19	*SIGLEC14*	78.8
cg17976473	10	*DMBT1*	78.8
cg23954274	10	*NA*	78.8
cg01157718	6	*NA*	77.5
cg04937939	2	*NA*	77.5
cg05546241	6	*NA*	77.5
cg06012695	6	*NA*	77.5
cg08208480	11	*BRSK2*	77.5
cg10099275	12	*PLCZ1*	77.5
cg10546252	22	*GSTT1*	77.5
cg11987751	1	*SLC35E2*	77.5
cg13739333	6	*HLA-DRB5*	77.5
cg14359894	20	*RASSF2*	77.5
cg15244290	6	*GUCA1A*	77.5
cg15700429	6	*HLA-DRB6*	77.5
cg16661157	17	*PRKCA*	77.5
cg20336016	19	*NA*	77.5
cg20968290	6	*CDKAL1*	77.5
cg23007425	14	*NA*	77.5
cg24565820	22	*LOC391322*	77.5
cg25120325	10	*PNLIPRP2*	77.5
cg27362989	6	*HLA-DRB5*	77.5
cg01253711	4	*NA*	76.3
cg14075454	6	*HLA-DRB1*	76.3
cg15253861	6	*HLA-DPA1*	76.3
cg15654485	6	*HLA-DRB6*	76.3
cg18428180	6	*KIAA0319*	76.3
cg18500967	7	*NA*	76.3
cg18868178	10	*NA*	76.3
cg18938907	1	*GSTM1*	76.3
cg20908200	20	*SNAP25-AS1*	76.3
cg21741515	3	*TM4SF19-TCTEX1D2*	76.3
cg23536255	6	*NA*	76.3
cg26430986	5	*GABRP*	76.3
cg01710670	16	*NA*	75.0
cg05919324	17	*RPH3AL*	75.0
cg07119157	1	*TNFRSF18*	75.0
cg07777042	8	*NA*	75.0
cg12015991	6	*HLA-DRB5*	75.0
cg12182903	4	*NA*	75.0
cg14035247	4	*NA*	75.0
cg15410402	X	*KRBOX4*	75.0
cg15680603	6	*HLA-DPA1*	75.0
cg17421046	6	*HLA-DQA1*	75.0
cg19516921	6	*HLA-DRB5*	75.0
cg00013655	16	*PIGQ*	73.8
cg03296304	11	*DEAF1*	73.8
cg04277055	4	*NA*	73.8
cg06398883	13	*NA*	73.8
cg06573709	6	*NA*	73.8
cg10662395	19	*HCN2*	73.8
cg13312387	2	*NMUR1*	73.8

cg16997375	22	*GSTT1*	73.8
cg20311868	7	*WBSCR27*	73.8
cg20593128	X	*NA*	73.8
cg02126896	11	*NA*	72.5
cg04777551	6	*HLA-DQB1*	72.5
cg06559318	6	*HLA-DRB6*	72.5
cg07774765	5	*BTNL9*	72.5
cg08708231	11	*OPCML*	72.5
cg14740251	19	*NA*	72.5
cg15293181	2	*SNTG2*	72.5
cg00534215	16	*ACSF3*	71.3
cg00814186	17	*NA*	71.3
cg01474688	2	*SP3*	71.3
cg03216697	6	*HLA-C*	71.3
cg04649587	8	*TDH*	71.3
cg06180910	22	*GSTT1*	71.3
cg11834635	4	*ZFYVE28*	71.3
cg12846737	6	*NA*	71.3
cg14294953	9	*MIR4289*	71.3
cg16514085	6	*HLA-DRB1*	71.3
cg19976037	17	*CCDC57*	71.3
cg20945531	6	*FLJ34503*	71.3
cg23905789	6	*HLA-DRB1*	71.3
cg24205048	19	*SIGLEC5*	71.3
cg24238852	22	*GSTT1*	71.3
cg01445689	7	*WDR60*	70.0
cg02145102	6	*NA*	70.0
cg07258408	2	*TSSC1*	70.0
cg07642767	10	*NA*	70.0
cg08759036	X	*OCRL*	70.0
cg10032131	11	*SLC43A1*	70.0
cg14744161	6	*DSE*	70.0
cg18021992	20	*ITPA*	70.0
cg19032705	5	*SH3PXD2B*	70.0
cg20064778	22	*GSTT1*	70.0
cg22956547	19	*ZNF155*	70.0
cg23505044	17	*NTN1*	70.0
cg25664050	6	*HLA-DRB6*	70.0
cg27129755	5	*GABRP*	70.0
cg03959986	17	*SRR*	68.8
cg04824771	22	*LOC391322*	68.8
cg04953552	6	*NA*	68.8
cg10995422	6	*HLA-DRB6*	68.8
cg11478607	22	*GSTT1*	68.8
cg17831170	6	*NA*	68.8
cg18576210	6	*HIST1H2AB*	68.8
cg01707391	16	*NA*	67.5
cg01744396	22	*GSTT1*	67.5
cg06236987	6	*FLJ34503*	67.5
cg08219170	22	*GSTT1*	67.5
cg08702834	9	*DMRT3*	67.5
cg11317459	13	*MIPEPP3*	67.5

cg11849573	7	*NA*	67.5
cg13004509	11	*CCDC67*	67.5
cg13365400	Y	*TTTY4C*	67.5
cg14310877	6	*NA*	67.5
cg15048554	12	*BTBD11*	67.5
cg16699528	7	*PVRIG*	67.5
cg17724121	11	*HBE1*	67.5
cg02018764	8	*NA*	66.3
cg03965138	19	*SIGLEC5*	66.3
cg05208607	16	*TLDC1*	66.3
cg05708441	2	*NA*	66.3
cg10506445	1	*NR5A2*	66.3
cg22666875	22	*GSTTP1*	66.3
cg26709433	6	*NA*	66.3
cg27627215	8	*NA*	66.3
cg01500431	22	*GSTTP2*	65.0
cg01957799	22	*GSTT1*	65.0
cg02943303	2	*TXNDC9*	65.0
cg13874759	17	*ENPP7*	65.0
cg16808927	16	*WDR90*	65.0
cg26881268	11	*NA*	65.0
cg00259849	8	*CSMD1*	63.8
cg05380919	22	*GSTT1*	63.8
cg22681945	1	*NA*	63.8
cg24336839	22	*GSTT1*	63.8
cg04926385	8	*NA*	62.5
cg15254640	22	*GSTT1*	62.5
cg00873648	6	*NA*	61.3
cg03007623	19	*CDKN2D*	61.3
cg07225509	5	*GALNT10*	61.3
cg11791288	1	*LOC284551*	61.3
cg20060160	1	*NMNAT2*	61.3
cg10804726	17	*DNAI2*	60.0
cg00256329	17	*NXN*	58.8
cg12546355	7	*NA*	58.8
cg22653957	2	*ALPPL2*	58.8
cg24098252	12	*GPR133*	58.8
cg02902672	6	*HLA-DQB1*	57.5
cg10385522	6	*HLA-DRB1*	57.5
cg11866463	21	*RCAN1*	57.5
cg19648023	2	*MIR548N*	56.3
cg27156510	17	*B9D1*	56.3
cg05419812	6	*NA*	55.0
cg00303108	X	*RBM41*	53.8
cg09476440	1	*KIAA1324*	53.8
cg17251423	5	*NA*	53.8
cg14117320	11	*PLEKHA7*	52.5
cg12280242	4	*NA*	51.3
cg09307193	14	*ACTN1*	50.0
cg22764591	12	*LOC100506844*	47.5
cg18845950	6	*NA*	46.3
cg05341252	6	*HLA-DQB1*	45.0

cg23699809	8	*INTS9*	43.8
cg19235801	10	*NA*	42.5
cg01550828	3	*RNF168*	41.3
cg07811112	15	*NA*	40.0
cg00749520	19	*TMEM238*	32.5
cg15544633	2	*LIPT1*	32.5
cg25985455	20	*PSMA7*	31.3
cg24834873	5	*ANKRD34B*	16.3

[a]Probe ID of the Infinium HumanMethylation 450 BeadChip. [b]*P*-values for detection of a signal above the background >0.01. NA, not annotated (designed for the intergenic regions). Since low call proportions may have been attributable to polymorphism at the probe CpG sites, the above 681 probes were excluded from the present assay.

Supplementary Table S4 The 219 genes showing significant inverse correlations between DNA methylation and mRNA expression levels (correlation coefficient [r] < -0.25 and *P* < 0.05) using The Cancer Genome Atlas database (https://cancergenome.nih.gov)

Gene Symbol	Probe ID[a]	CpG type[b]	Annotation[c]	Type of cancer[d]	R	*P*
A2LD1	cg25710107	S_Shelf	TSS1500	LIHC	-0.312	< 0.001
				KIRC	-0.284	< 0.001
ABCC3	cg27222669	N_Shore	TSS1500	STAD	-0.389	< 0.001
				LUAD	-0.510	< 0.001
				KIRC	-0.446	< 0.001
ADAM32	cg22848598	CpG island	TSS200	UCEC	-0.283	< 0.001
ADCY5	cg04138502	CpG island	TSS200	STAD	-0.254	< 0.001
				LUAD	-0.308	< 0.001
				LIHC	-0.315	< 0.001
				KIRC	-0.493	< 0.001
ADIPOR1	cg17218466	S_Shore	TSS1500	STAD	-0.362	< 0.001
				LUAD	-0.384	< 0.001
				UCEC	-0.305	< 0.001
AGXT2L2	cg20721320	S_Shore	TSS1500	KIRC	-0.260	< 0.001
AKAP8L	cg13640414	S_Shore	TSS1500	LIHC	-0.276	< 0.001
				UCEC	-0.289	< 0.001
	cg25533247	S_Shore	TSS1500	LIHC	-0.271	< 0.001
ALDH1L2	cg16204509	S_Shore	TSS1500	LUAD	-0.252	< 0.001
ALS2CR11	cg11052143	S_Shore	TSS200	STAD	-0.423	< 0.001
				LUAD	-0.401	< 0.001
				LIHC	-0.339	< 0.001
				UCEC	-0.342	< 0.001
AMPD3	cg07329251	S_Shelf	TSS200	LUAD	-0.294	< 0.001
				KIRC	-0.438	< 0.001
	cg19132462	S_Shelf	TSS200	KIRC	-0.483	< 0.001
	cg01116568	S_Shelf	TSS200	KIRC	-0.455	< 0.001
ANK1	cg26984624	S_Shore	TSS1500	KIRC	-0.360	< 0.001
	cg04153489	CpG island	TSS1500	KIRC	-0.355	< 0.001
ANKRD40	cg20807852	S_Shore	TSS1500	UCEC	-0.398	< 0.001
ARHGAP9	cg08339023	S_Shelf	TSS200	STAD	-0.263	< 0.001
				LUAD	-0.402	< 0.001
				KIRC	-0.427	< 0.001
	cg03214715	S_Shelf	TSS1500	KIRC	-0.365	< 0.001
ASCL2	cg05309948	CpG island	TSS1500	LUAD	-0.259	< 0.001
ATG16L1	cg23339656	N_Shore	TSS1500	KIRC	-0.265	< 0.001
B3GNT3	cg23771661	N_Shore	TSS1500	STAD	-0.365	< 0.001
BIRC7	cg22843950	N_Shelf	TSS200	KIRC	-0.375	< 0.001
BRD1	cg16001335	N_Shore; N_Shore; S_Shelf	TSS1500	LUAD	-0.372	< 0.001
C10orf72	cg06484553	S_Shore	TSS1500	KIRC	-0.289	< 0.001
	cg08427977	S_Shore	TSS1500	KIRC	-0.267	< 0.001
C14orf180	cg02868468	S_Shelf	TSS1500	KIRC	-0.310	< 0.001
C14orf73	cg09380688	N_Shelf	TSS200	LIHC	-0.612	< 0.001
C17orf101	cg24998110	S_Shelf; S_Shore	TSS1500	KIRC	-0.264	< 0.001
C17orf104	cg08496953	CpG island	TSS1500	STAD	-0.341	< 0.001
				LUAD	-0.264	< 0.001
				UCEC	-0.269	< 0.001
	cg22605919	CpG island	TSS1500	STAD	-0.339	< 0.001
				LUAD	-0.268	< 0.001
				UCEC	-0.279	< 0.001
C1orf200	cg12354861	S_Shore	TSS200	KIRC	-0.464	< 0.001
C1orf66	cg02833725	N_Shore	TSS1500	STAD	-0.295	< 0.001
C1orf70	cg15487867	S_Shelf; N_Shore; S_Shore	TSS200	STAD	-0.360	< 0.001
				UCEC	-0.386	< 0.001
C8orf38	cg27127492	N_Shore	TSS1500	LUAD	-0.320	< 0.001
				LIHC	-0.347	< 0.001

C8orf84	cg19518093	S_Shore	TSS1500	UCEC	-0.331	< 0.001
CACNA1B	cg05863502	CpG island	TSS1500	UCEC	-0.269	< 0.001
CAD	cg09947274	N_Shore	TSS1500	LUAD	-0.290	< 0.001
				LIHC	-0.404	< 0.001
				KIRC	-0.368	< 0.001
CARD14	cg06209035	N_Shelf	TSS200	STAD	-0.296	< 0.001
				LUAD	-0.501	< 0.001
				KIRC	-0.357	< 0.001
CARNS1	cg25414605	N_Shore	TSS1500	UCEC	-0.329	< 0.001
CCDC24	cg22816909	N_Shore	TSS1500	STAD	-0.277	< 0.001
				LUAD	-0.291	< 0.001
CCHCR1	cg24876187	S_Shore	TSS1500	LIHC	-0.261	< 0.001
				KIRC	-0.253	< 0.001
	cg23238834	S_Shore	TSS1500	KIRC	-0.271	< 0.001
CD274	cg15837913	N_Shore	TSS1500	UCEC	-0.254	< 0.001
CD44	cg20971158	N_Shore	TSS1500	KIRC	-0.263	< 0.001
CDKL2	cg03757145	CpG island	TSS200	UCEC	-0.283	< 0.001
	cg14263942	CpG island	TSS200	UCEC	-0.282	< 0.001
CHMP4C	cg07583137	N_Shore	TSS1500	STAD	-0.569	< 0.001
				LUAD	-0.471	< 0.001
				LIHC	-0.390	< 0.001
				KIRC	-0.306	< 0.001
				UCEC	-0.456	< 0.001
CIITA	cg08985333	N_Shore	TSS200	STAD	-0.276	< 0.001
				KIRC	-0.314	< 0.001
CKLF	cg08793208	N_Shore; S_Shore	TSS1500	LIHC	-0.355	< 0.001
CLDN4	cg14826417	N_Shore	TSS1500	STAD	-0.447	< 0.001
				LUAD	-0.350	< 0.001
				LIHC	-0.251	< 0.001
				KIRC	-0.289	< 0.001
				UCEC	-0.452	< 0.001
CMTM2	cg05546863	CpG island	TSS200	LUAD	-0.368	< 0.001
	cg07153665	CpG island	TSS200	LUAD	-0.362	< 0.001
CMTM4	cg02420395	S_Shore	TSS1500	KIRC	-0.336	< 0.001
CNO	cg20644952	N_Shore	TSS1500	STAD	-0.301	< 0.001
				LUAD	-0.408	< 0.001
COX10	cg20170777	N_Shore	TSS1500	STAD	-0.262	< 0.001
				LUAD	-0.302	< 0.001
COX8C	cg21149595	CpG island	TSS200	UCEC	-0.317	< 0.001
DBF4	cg04330806	N_Shore	TSS1500	STAD	-0.259	< 0.001
				LIHC	-0.307	< 0.001
				UCEC	-0.315	< 0.001
DBNDD1	cg02872476	N_Shelf; S_Shore	TSS1500	STAD	-0.475	< 0.001
				LUAD	-0.477	< 0.001
				LIHC	-0.511	< 0.001
				KIRC	-0.397	< 0.001
				UCEC	-0.589	< 0.001
DCAF4L2	cg02310286	CpG island	TSS200	STAD	-0.337	< 0.001
				LUAD	-0.277	< 0.001
				LIHC	-0.435	< 0.001
				KIRC	-0.495	< 0.001
				UCEC	-0.386	< 0.001
	cg21181391	CpG island	TSS200	STAD	-0.255	< 0.001
				LIHC	-0.374	< 0.001
				KIRC	-0.572	< 0.001
				UCEC	-0.344	< 0.001
	cg17703554	CpG island	TSS200	LIHC	-0.394	< 0.001
				KIRC	-0.575	< 0.001

				UCEC	-0.273	< 0.001
DERL1	cg21139496	S_Shore	TSS1500	UCEC	-0.253	< 0.001
DNASE2	cg15503081	N_Shelf; S_Shore	TSS1500	KIRC	-0.495	< 0.001
				UCEC	-0.309	< 0.001
DYDC1	cg12640394	CpG island	TSS200	LIHC	-0.255	< 0.001
E2F6	cg23214755	S_Shore	TSS1500	LUAD	-0.391	< 0.001
				LIHC	-0.324	< 0.001
	cg17726575	S_Shore	TSS1500	LUAD	-0.446	< 0.001
				LIHC	-0.314	< 0.001
EHBP1L1	cg14189782	hore; S_Shelf; CpG island; N_Sh	TSS1500	KIRC	-0.411	< 0.001
EPHX3	cg26010734	S_Shore; N_Shore	TSS200	STAD	-0.351	< 0.001
				LUAD	-0.313	< 0.001
				LIHC	-0.286	< 0.001
				KIRC	-0.257	< 0.001
				UCEC	-0.360	< 0.001
	cg18366919	S_Shore; CpG island	TSS1500	STAD	-0.346	< 0.001
				LUAD	-0.347	< 0.001
				LIHC	-0.282	< 0.001
				KIRC	-0.275	< 0.001
				UCEC	-0.370	< 0.001
FAM110A	cg01687401	S_Shelf; N_Shore	TSS1500	UCEC	-0.304	< 0.001
FAM111A	cg15723195	S_Shore	TSS1500	STAD	-0.399	< 0.001
				LIHC	-0.316	< 0.001
				UCEC	-0.304	< 0.001
FAM164C	cg00327772	N_Shore	TSS1500	STAD	-0.287	< 0.001
				KIRC	-0.449	< 0.001
FCHO1	cg11737334	S_Shelf	TSS200	KIRC	-0.348	< 0.001
FLJ43663	cg05632420	S_Shore; N_Shore	TSS200	STAD	-0.274	< 0.001
				LUAD	-0.315	< 0.001
FZD6	cg09020649	N_Shore	TSS200	LUAD	-0.269	< 0.001
				UCEC	-0.327	< 0.001
GALNT14	cg18624059	S_Shore	TSS1500	KIRC	-0.286	< 0.001
GGCT	cg21491201	S_Shore	TSS1500	STAD	-0.274	< 0.001
				LUAD	-0.351	< 0.001
				LIHC	-0.262	< 0.001
				KIRC	-0.290	< 0.001
GON4L	cg00504075	N_Shore; N_Shelf	TSS1500	LUAD	-0.294	< 0.001
				LIHC	-0.270	< 0.001
H1F0	cg15006681	N_Shelf; N_Shore	TSS1500	STAD	-0.334	< 0.001
				LUAD	-0.368	< 0.001
				LIHC	-0.440	< 0.001
				UCEC	-0.318	< 0.001
HDAC11	cg19779799	CpG island; N_Shore	TSS1500	STAD	-0.275	< 0.001
	cg05987698	CpG island; N_Shore	TSS1500	STAD	-0.250	< 0.001
HIST1H2BH	cg02578368	CpG island; N_Shore; S_Shelf	TSS1500	STAD	-0.324	< 0.001
				LUAD	-0.265	< 0.001
HIST1H3H	cg01330280	N_Shore; S_Shore	TSS1500	UCEC	-0.268	< 0.001
HK2	cg05595692	N_Shore	TSS1500	KIRC	-0.424	< 0.001
HMGN2	cg27425314	N_Shore	TSS1500	LIHC	-0.259	< 0.001
HNRNPF	cg08343600	S_Shore	TSS1500	STAD	-0.522	< 0.001
				LUAD	-0.452	< 0.001
				UCEC	-0.417	< 0.001
HOXA10	cg14188840	N_Shelf; S_Shore	TSS1500	STAD	-0.612	< 0.001
				UCEC	-0.678	< 0.001
	cg14625175	CpG island	TSS200	STAD	-0.454	< 0.001
				KIRC	-0.279	< 0.001
				UCEC	-0.427	< 0.001
HOXA4	cg16651126	CpG island	TSS200	STAD	-0.302	< 0.001

				LUAD	-0.315	< 0.001
				LIHC	-0.412	< 0.001
				UCEC	-0.409	< 0.001
	cg11015251	CpG island	TSS200	STAD	-0.281	< 0.001
				LUAD	-0.327	< 0.001
				LIHC	-0.422	< 0.001
				UCEC	-0.410	< 0.001
HOXD8	cg14473102	S_Shore; CpG island	TSS200	STAD	-0.429	< 0.001
				LUAD	-0.297	< 0.001
				KIRC	-0.256	< 0.001
				UCEC	-0.254	< 0.001
HTATIP2	cg18788940	N_Shore	TSS200	STAD	-0.429	< 0.001
				LIHC	-0.395	< 0.001
				KIRC	-0.423	< 0.001
				UCEC	-0.305	< 0.001
IER5	cg02661372	N_Shore	TSS1500	LUAD	-0.275	< 0.001
INA	cg23642747	CpG island	TSS1500	LUAD	-0.261	< 0.001
				UCEC	-0.305	< 0.001
INPP5D	cg06272010	N_Shore	TSS1500	STAD	-0.488	< 0.001
				LUAD	-0.473	< 0.001
				KIRC	-0.477	< 0.001
				UCEC	-0.474	< 0.001
	cg02935132	N_Shore	TSS1500	STAD	-0.487	< 0.001
				LUAD	-0.430	< 0.001
				KIRC	-0.376	< 0.001
				UCEC	-0.457	< 0.001
	cg00438740	N_Shore	TSS200	STAD	-0.496	< 0.001
				LUAD	-0.406	< 0.001
				KIRC	-0.258	< 0.001
				UCEC	-0.473	< 0.001
	cg02788013	N_Shore	TSS200	STAD	-0.391	< 0.001
				LUAD	-0.326	< 0.001
				UCEC	-0.280	< 0.001
ITGA4	cg05246303	N_Shore	TSS1500	STAD	-0.273	< 0.001
ITGA6	cg20795401	N_Shore	TSS1500	STAD	-0.286	< 0.001
ITGA7	cg25814752	N_Shelf	TSS1500	LIHC	-0.332	< 0.001
				KIRC	-0.296	< 0.001
KCNK9	cg21415530	CpG island	TSS1500	KIRC	-0.257	< 0.001
				UCEC	-0.263	< 0.001
KRT19	cg12491114	S_Shore	TSS1500	STAD	-0.372	< 0.001
				KIRC	-0.522	< 0.001
	cg03550233	S_Shore	TSS1500	STAD	-0.297	< 0.001
				KIRC	-0.440	< 0.001
L3MBTL	cg22330467	N_Shore	TSS200	LUAD	-0.319	< 0.001
LAG3	cg04153135	N_Shore	TSS1500	LUAD	-0.274	< 0.001
LFNG	cg05829046	N_Shore	TSS200	KIRC	-0.322	< 0.001
	cg12497581	N_Shore	TSS1500	KIRC	-0.251	< 0.001
LOC100133545	cg07397493	N_Shelf	TSS200	LIHC	-0.295	< 0.001
	cg08273750	N_Shelf	TSS200	LIHC	-0.255	< 0.001
LOC387647	cg11515215	N_Shore	TSS1500	LIHC	-0.327	< 0.001
LOC441601	cg25318278	S_Shore	TSS200	LUAD	-0.262	< 0.001
				LIHC	-0.295	< 0.001
LRRK1	cg25331703	CpG island	TSS200	UCEC	-0.295	< 0.001
LRRN1	cg04426311	CpG island	TSS200	STAD	-0.336	< 0.001
LTB	cg13335388	S_Shore	TSS200	UCEC	-0.358	< 0.001
LUC7L3	cg04604142	N_Shore	TSS1500	LUAD	-0.289	< 0.001
				LIHC	-0.252	< 0.001
LYNX1	cg23180489	S_Shore	TSS200	LUAD	-0.350	< 0.001

	cg19021188	S_Shore	TSS200	LUAD	-0.406	< 0.001
	cg08090164	S_Shore	TSS200	LUAD	-0.408	< 0.001
LYPD1	cg20273260	S_Shore	TSS200	LIHC	-0.271	< 0.001
MAP6D1	cg10347032	CpG island	TSS1500	STAD	-0.269	< 0.001
MAPK13	cg05859264	N_Shore	TSS1500	STAD	-0.510	< 0.001
				LUAD	-0.328	< 0.001
				LIHC	-0.377	< 0.001
				KIRC	-0.418	< 0.001
				UCEC	-0.465	< 0.001
MELK	cg14279528	N_Shore	TSS1500	STAD	-0.332	< 0.001
				LIHC	-0.336	< 0.001
				UCEC	-0.257	< 0.001
MEST	cg20148994	N_Shore	TSS1500	STAD	-0.414	< 0.001
				LUAD	-0.520	< 0.001
	cg22115706	N_Shore	TSS1500	STAD	-0.387	< 0.001
				LUAD	-0.456	< 0.001
	cg07683330	N_Shore	TSS1500	STAD	-0.369	< 0.001
				LUAD	-0.472	< 0.001
MICB	cg08009669	N_Shore; S_Shelf	TSS1500	LIHC	-0.322	< 0.001
	cg20240154	N_Shore; S_Shelf	TSS1500	LIHC	-0.274	< 0.001
				KIRC	-0.338	< 0.001
MOV10L1	cg10828284	N_Shore; CpG island	TSS200	STAD	-0.474	< 0.001
				UCEC	-0.456	< 0.001
MPV17L	cg09294492	N_Shore	TSS1500	STAD	-0.304	< 0.001
MRPS36	cg15556672	N_Shore	TSS1500	LUAD	-0.257	< 0.001
MTMR7	cg12296772	CpG island	TSS200	UCEC	-0.361	< 0.001
MYO19	cg02097479	S_Shore	TSS1500	STAD	-0.402	< 0.001
				LUAD	-0.374	< 0.001
				LIHC	-0.261	< 0.001
				UCEC	-0.357	< 0.001
MYO5C	cg27633342	S_Shore	TSS1500	STAD	-0.362	< 0.001
				LUAD	-0.338	< 0.001
				LIHC	-0.365	< 0.001
				UCEC	-0.306	< 0.001
NCALD	cg19308132	S_Shore	TSS1500	LUAD	-0.359	< 0.001
				UCEC	-0.443	< 0.001
NCAPH	cg03913456	N_Shore	TSS1500	STAD	-0.274	< 0.001
NCOA6	cg17129400	S_Shore	TSS1500	STAD	-0.496	< 0.001
				LUAD	-0.318	< 0.001
				UCEC	-0.322	< 0.001
	cg10743062	S_Shore	TSS1500	STAD	-0.473	< 0.001
				LUAD	-0.351	< 0.001
				LIHC	-0.274	< 0.001
				UCEC	-0.302	< 0.001
NCRNA00200	cg00666074	N_Shore	TSS1500	STAD	-0.308	< 0.001
NEFM	cg17078116	CpG island	TSS200	STAD	-0.432	< 0.001
				LIHC	-0.254	< 0.001
				UCEC	-0.475	< 0.001
NOX4	cg19981409	S_Shore	TSS1500	LUAD	-0.287	< 0.001
NRN1L	cg10574499	CpG island	TSS200	LUAD	-0.325	< 0.001
	cg09756419	CpG island	TSS200	LUAD	-0.310	< 0.001
	cg01068601	CpG island	TSS200	LUAD	-0.287	< 0.001
	cg02308079	CpG island	TSS200	LUAD	-0.278	< 0.001
ODZ4	cg00908927	S_Shore	TSS1500	KIRC	-0.333	< 0.001
PARD6B	cg03812837	N_Shore	TSS1500	STAD	-0.386	< 0.001
				KIRC	-0.398	< 0.001
				UCEC	-0.380	< 0.001
PARP9	cg24087515	S_Shore	TSS1500	UCEC	-0.430	< 0.001

PCDHB8	cg09052108	S_Shelf; N_Shore	TSS200	STAD	-0.378	< 0.001
				LUAD	-0.337	< 0.001
				KIRC	-0.454	< 0.001
				UCEC	-0.442	< 0.001
PCDHGA11	cg13951490	hore; N_Shore; N_Shelf; CpG isl	TSS1500	STAD	-0.418	< 0.001
				UCEC	-0.515	< 0.001
PFDN6	cg27355501	N_Shore	TSS1500	STAD	-0.252	< 0.001
				LIHC	-0.252	< 0.001
PGAP2	cg19654882	N_Shore	TSS200	UCEC	-0.275	< 0.001
PIK3CG	cg24384195	N_Shelf	TSS1500	STAD	-0.426	< 0.001
				LUAD	-0.338	< 0.001
				KIRC	-0.337	< 0.001
				UCEC	-0.294	< 0.001
	cg13214190	N_Shelf	TSS200	LUAD	-0.338	< 0.001
				KIRC	-0.339	< 0.001
				UCEC	-0.329	< 0.001
	cg11982525	N_Shelf	TSS1500	LUAD	-0.290	< 0.001
PLEKHN1	cg12663811	CpG island	TSS200	LIHC	-0.346	< 0.001
				KIRC	-0.313	< 0.001
				UCEC	-0.380	< 0.001
PLLP	cg10343787	S_Shore	TSS1500	LUAD	-0.266	< 0.001
PMEPA1	cg08082355	S_Shore	TSS1500	STAD	-0.321	< 0.001
				KIRC	-0.494	< 0.001
	cg20208990	S_Shore	TSS1500	STAD	-0.299	< 0.001
				KIRC	-0.478	< 0.001
	cg07143805	S_Shore	TSS200	KIRC	-0.379	< 0.001
PNMAL1	cg14750269	S_Shore	TSS1500	LUAD	-0.416	< 0.001
				UCEC	-0.342	< 0.001
PPP1R14A	cg02571816	CpG island	TSS200	STAD	-0.305	< 0.001
				LUAD	-0.347	< 0.001
				LIHC	-0.299	< 0.001
	cg09515953	CpG island	TSS200	STAD	-0.304	< 0.001
				LUAD	-0.344	< 0.001
	cg22557662	CpG island	TSS200	STAD	-0.299	< 0.001
				LUAD	-0.324	< 0.001
PPP2R3A	cg07038400	N_Shore	TSS1500	STAD	-0.302	< 0.001
				LIHC	-0.300	< 0.001
	cg23194766	N_Shore	TSS1500	STAD	-0.300	< 0.001
	cg04907151	N_Shore	TSS1500	STAD	-0.294	< 0.001
				LIHC	-0.268	< 0.001
PRAME	cg22871485	S_Shore	TSS200	STAD	-0.303	< 0.001
				LUAD	-0.390	< 0.001
				LIHC	-0.255	< 0.001
				KIRC	-0.351	< 0.001
				UCEC	-0.522	< 0.001
	cg00372774	S_Shore	TSS1500	UCEC	-0.520	< 0.001
PRC1	cg01407062	S_Shore	TSS1500	STAD	-0.497	< 0.001
				LUAD	-0.370	< 0.001
				LIHC	-0.418	< 0.001
				UCEC	-0.406	< 0.001
PRF1	cg23059461	S_Shelf	TSS200	LUAD	-0.271	< 0.001
				KIRC	-0.513	< 0.001
	cg12336290	S_Shelf	TSS200	LUAD	-0.256	< 0.001
				KIRC	-0.403	< 0.001
PRR16	cg06526721	N_Shore; N_Shore	TSS1500	KIRC	-0.250	< 0.001
	cg08981702	N_Shore; N_Shore	TSS1500	UCEC	-0.260	< 0.001
PSMA8	cg11858305	N_Shore	TSS1500	LUAD	-0.272	< 0.001
PSMD2	cg06306006	S_Shore	TSS200	LUAD	-0.288	< 0.001

				UCEC	-0.440	< 0.001
PSMD6	cg01808508	S_Shore	TSS1500	UCEC	-0.311	< 0.001
PTCH1	cg26878949	CpG island	TSS1500	KIRC	-0.412	< 0.001
RAB15	cg14944269	S_Shore	TSS1500	STAD	-0.392	< 0.001
				LUAD	-0.255	< 0.001
RAP1GAP	cg17184245	S_Shore; S_Shore	TSS1500	LUAD	-0.334	< 0.001
RASSF10	cg08876434	CpG island	TSS1500	STAD	-0.323	< 0.001
	cg05095158	CpG island	TSS200	STAD	-0.289	< 0.001
RBM24	cg15168816	S_Shore	TSS1500	STAD	-0.259	< 0.001
				LIHC	-0.340	< 0.001
				KIRC	-0.278	< 0.001
RCC1	cg25596287	N_Shore	TSS1500	STAD	-0.318	< 0.001
				LUAD	-0.277	< 0.001
				LIHC	-0.257	< 0.001
				KIRC	-0.379	< 0.001
				UCEC	-0.266	< 0.001
RHOU	cg03145930	N_Shore	TSS1500	LUAD	-0.279	< 0.001
RIMS2	cg20129213	CpG island	TSS1500	LUAD	-0.252	< 0.001
RIN1	cg08995609	S_Shore	TSS200	STAD	-0.395	< 0.001
				LUAD	-0.277	< 0.001
				LIHC	-0.255	< 0.001
				KIRC	-0.310	< 0.001
				UCEC	-0.291	< 0.001
	cg14391855	S_Shore	TSS200	LUAD	-0.279	< 0.001
				LIHC	-0.258	< 0.001
				KIRC	-0.373	< 0.001
RIPK3	cg27599271	S_Shore	TSS200	STAD	-0.382	< 0.001
				KIRC	-0.275	< 0.001
				UCEC	-0.492	< 0.001
RNF212	cg23462514	CpG island	TSS1500	STAD	-0.480	< 0.001
				LUAD	-0.424	< 0.001
				KIRC	-0.340	< 0.001
				UCEC	-0.412	< 0.001
	cg12414557	CpG island	TSS200	STAD	-0.392	< 0.001
				LUAD	-0.357	< 0.001
				KIRC	-0.284	< 0.001
				UCEC	-0.391	< 0.001
RNF220	cg21578207	N_Shore	TSS1500	LIHC	-0.296	< 0.001
				KIRC	-0.252	< 0.001
RPS6KA1	cg22512670	N_Shore	TSS1500	STAD	-0.303	< 0.001
				UCEC	-0.461	< 0.001
RRBP1	cg26447697	S_Shore	TSS1500	STAD	-0.299	< 0.001
S100A6	cg08106792	S_Shore	TSS1500	STAD	-0.267	< 0.001
				KIRC	-0.411	< 0.001
				UCEC	-0.264	< 0.001
SAAL1	cg05093315	S_Shore	TSS1500	LIHC	-0.330	< 0.001
				KIRC	-0.254	< 0.001
SALL3	cg05080154	CpG island	TSS1500	KIRC	-0.254	< 0.001
SAMD10	cg05929574	N_Shore; S_Shore	TSS1500	LIHC	-0.304	< 0.001
				UCEC	-0.305	< 0.001
SAP18	cg23082003	N_Shore	TSS1500	STAD	-0.329	< 0.001
				LUAD	-0.267	< 0.001
SCLT1	cg05812836	S_Shore	TSS1500	LIHC	-0.265	< 0.001
SCN8A	cg18426142	N_Shore	TSS1500	STAD	-0.276	< 0.001
				LUAD	-0.301	< 0.001
				UCEC	-0.295	< 0.001
SDF2	cg07042338	S_Shore	TSS1500	LIHC	-0.251	< 0.001
SDR16C5	cg05305140	S_Shore	TSS1500	LUAD	-0.285	< 0.001

	cg18140857	S_Shore	TSS1500	LUAD	-0.289	< 0.001
SEMA4F	cg26801943	N_Shore	TSS1500	LIHC	-0.327	< 0.001
				KIRC	-0.264	< 0.001
SFN	cg06720467	N_Shore; N_Shore	TSS200	LIHC	-0.417	< 0.001
	cg21950166	N_Shore; N_Shore	TSS1500	LIHC	-0.461	< 0.001
SH3BP1	cg20170271	N_Shore	TSS1500	STAD	-0.424	< 0.001
				KIRC	-0.449	< 0.001
SHE	cg18081940	CpG island	TSS1500	STAD	-0.301	< 0.001
				KIRC	-0.301	< 0.001
				UCEC	-0.254	< 0.001
SHPK	cg17342247	S_Shore	TSS1500	LUAD	-0.254	< 0.001
SLC13A5	cg13545212	CpG island	TSS1500	LIHC	-0.251	< 0.001
SLC16A1	cg26162147	S_Shore; S_Shore	TSS1500	STAD	-0.253	< 0.001
				LUAD	-0.396	< 0.001
				KIRC	-0.319	< 0.001
				UCEC	-0.581	< 0.001
	cg07176692	S_Shelf; S_Shore	TSS1500	LUAD	-0.290	< 0.001
				KIRC	-0.351	< 0.001
				UCEC	-0.539	< 0.001
SLC22A18AS	cg08999895	S_Shelf	TSS1500	STAD	-0.358	< 0.001
				LIHC	-0.354	< 0.001
SLC25A36	cg03586379	CpG island	TSS200	LIHC	-0.381	< 0.001
SLC6A19	cg26711638	N_Shore	TSS1500	KIRC	-0.277	< 0.001
	cg21492882	CpG island	TSS200	KIRC	-0.254	< 0.001
SLC6A20	cg13719314	S_Shore	TSS1500	LUAD	-0.272	< 0.001
SLMO1	cg15262515	N_Shore	TSS1500	LUAD	-0.344	< 0.001
SMYD5	cg01329511	N_Shore	TSS1500	STAD	-0.318	< 0.001
				LUAD	-0.266	< 0.001
				LIHC	-0.435	< 0.001
SNRPD1	cg22215678	N_Shore	TSS1500	STAD	-0.385	< 0.001
				LUAD	-0.336	< 0.001
				LIHC	-0.256	< 0.001
SORD	cg06424894	N_Shore	TSS1500	UCEC	-0.272	< 0.001
SPAG6	cg18247055	CpG island; S_Shelf	TSS200	UCEC	-0.375	< 0.001
	cg23016129	CpG island; S_Shelf	TSS200	UCEC	-0.346	< 0.001
	cg12377139	CpG island; S_Shelf	TSS200	UCEC	-0.363	< 0.001
SPI1	cg03301240	S_Shore	TSS1500	KIRC	-0.306	< 0.001
SPO11	cg13888886	N_Shore	TSS200	LUAD	-0.281	< 0.001
SPSB2	cg09080114	S_Shore	TSS1500	STAD	-0.257	< 0.001
				LIHC	-0.260	< 0.001
SSB	cg23497644	N_Shore	TSS1500	STAD	-0.412	< 0.001
				LUAD	-0.302	< 0.001
STAG3	cg13210467	CpG island	TSS1500	STAD	-0.336	< 0.001
				LUAD	-0.408	< 0.001
				UCEC	-0.313	< 0.001
	cg04778012	CpG island	TSS1500	STAD	-0.331	< 0.001
				LUAD	-0.395	< 0.001
				UCEC	-0.315	< 0.001
	cg10084644	CpG island	TSS1500	STAD	-0.294	< 0.001
				LUAD	-0.316	< 0.001
				UCEC	-0.277	< 0.001
STX6	cg15084269	S_Shore; S_Shore	TSS1500	LIHC	-0.267	< 0.001
SUSD4	cg12307840	CpG island	TSS1500	KIRC	-0.335	< 0.001
	cg00172597	CpG island	TSS1500	KIRC	-0.302	< 0.001
	cg07605211	CpG island	TSS1500	KIRC	-0.281	< 0.001
SYCE1	cg14239307	N_Shelf; CpG island	TSS200	STAD	-0.444	< 0.001
				LUAD	-0.348	< 0.001
				LIHC	-0.528	< 0.001

				KIRC	-0.290	< 0.001
				UCEC	-0.440	< 0.001
TBX4	cg14823851	CpG island; S_Shelf	TSS1500	LUAD	-0.560	< 0.001
TBX6	cg14370448	N_Shelf; S_Shore	TSS1500	LIHC	-0.297	< 0.001
				KIRC	-0.260	< 0.001
TESC	cg01409207	S_Shore	TSS1500	LUAD	-0.315	< 0.001
TET1	cg23602092	N_Shore	TSS1500	KIRC	-0.269	< 0.001
THOC6	cg24652919	S_Shore; N_Shore	TSS1500	LIHC	-0.321	< 0.001
TMC6	cg24988684	S_Shore	TSS200	LUAD	-0.275	< 0.001
				UCEC	-0.404	< 0.001
TMEM51	cg22453656	N_Shore	TSS1500	STAD	-0.258	< 0.001
TNFRSF10A	cg22843797	CpG island	TSS1500	STAD	-0.271	< 0.001
				KIRC	-0.274	< 0.001
	cg23882019	CpG island	TSS1500	STAD	-0.258	< 0.001
				KIRC	-0.282	< 0.001
TRAF2	cg21236500	N_Shore	TSS1500	STAD	-0.323	< 0.001
				LUAD	-0.466	< 0.001
				LIHC	-0.530	< 0.001
				KIRC	-0.326	< 0.001
				UCEC	-0.311	< 0.001
TRIM4	cg02756107	S_Shore	TSS1500	UCEC	-0.371	< 0.001
	cg25591451	S_Shore	TSS1500	LUAD	-0.254	< 0.001
				UCEC	-0.267	< 0.001
TRPM5	cg16374193	S_Shelf	TSS1500	STAD	-0.268	< 0.001
TSPYL5	cg22319311	CpG island	TSS200	STAD	-0.688	< 0.001
				LUAD	-0.526	< 0.001
				LIHC	-0.351	< 0.001
				KIRC	-0.472	< 0.001
				UCEC	-0.733	< 0.001
TUBA1B	cg13709639	S_Shore	TSS1500	LIHC	-0.386	< 0.001
TUBB	cg05125809	N_Shore; N_Shore; S_Shore	TSS1500	UCEC	-0.282	< 0.001
	cg15677364	N_Shore; N_Shore; S_Shore	TSS1500	UCEC	-0.271	< 0.001
UBE2C	cg19197419	CpG island	TSS200	LIHC	-0.283	< 0.001
				UCEC	-0.287	< 0.001
UBE2V2	cg25612480	N_Shore	TSS1500	STAD	-0.339	< 0.001
				LUAD	-0.400	< 0.001
				UCEC	-0.329	< 0.001
USP21	cg18176482	N_Shore	TSS1500	STAD	-0.262	< 0.001
				LIHC	-0.329	< 0.001
				UCEC	-0.259	< 0.001
	cg01447427	N_Shore	TSS1500	LIHC	-0.322	< 0.001
	cg17017284	N_Shore	TSS1500	LIHC	-0.281	< 0.001
USP44	cg13879483	CpG island	TSS1500	STAD	-0.475	< 0.001
				LUAD	-0.358	< 0.001
				LIHC	-0.329	< 0.001
				KIRC	-0.365	< 0.001
				UCEC	-0.387	< 0.001
	cg22802813	CpG island	TSS200	STAD	-0.450	< 0.001
				LUAD	-0.317	< 0.001
				LIHC	-0.363	< 0.001
				KIRC	-0.376	< 0.001
				UCEC	-0.316	< 0.001
	cg07783282	CpG island	TSS1500	STAD	-0.390	< 0.001
				LUAD	-0.360	< 0.001
				LIHC	-0.281	< 0.001
				KIRC	-0.290	< 0.001
				UCEC	-0.396	< 0.001
UTP23	cg09305680	N_Shore	TSS1500	STAD	-0.321	< 0.001

				LUAD	-0.350	< 0.001
				LIHC	-0.309	< 0.001
VANGL2	cg13428480	CpG island	TSS200	STAD	-0.557	< 0.001
				LUAD	-0.494	< 0.001
				LIHC	-0.401	< 0.001
				KIRC	-0.388	< 0.001
VIM	cg10790685	N_Shore	TSS1500	KIRC	-0.462	< 0.001
VWDE	cg25431479	S_Shore	TSS1500	STAD	-0.273	< 0.001
				LUAD	-0.261	< 0.001
WBSCR26	cg17240725	N_Shelf	TSS200	STAD	-0.357	< 0.001
				KIRC	-0.295	< 0.001
				UCEC	-0.305	< 0.001
WSB2	cg01932247	S_Shore	TSS1500	LUAD	-0.264	< 0.001
				LIHC	-0.312	< 0.001
ZIC1	cg01227537	N_Shelf; CpG island	TSS200	STAD	-0.357	< 0.001
				UCEC	-0.373	< 0.001
	cg14456683	N_Shelf; CpG island	TSS200	STAD	-0.347	< 0.001
				UCEC	-0.355	< 0.001
ZIC5	cg00529958	CpG island	TSS200	UCEC	-0.411	< 0.001
ZNF132	cg11618529	CpG island	TSS1500	STAD	-0.300	< 0.001
				KIRC	-0.479	< 0.001
				UCEC	-0.497	< 0.001
ZNF14	cg19714957	CpG island	TSS200	STAD	-0.445	< 0.001
				LIHC	-0.506	< 0.001
ZNF154	cg01268824	S_Shore	TSS1500	STAD	-0.424	< 0.001
				LUAD	-0.527	< 0.001
				LIHC	-0.285	< 0.001
				KIRC	-0.355	< 0.001
				UCEC	-0.516	< 0.001
	cg03234186	S_Shore	TSS200	STAD	-0.423	< 0.001
				LUAD	-0.554	< 0.001
				LIHC	-0.277	< 0.001
				KIRC	-0.370	< 0.001
				UCEC	-0.521	< 0.001
ZNF215	cg04053776	N_Shore	TSS1500	STAD	-0.303	< 0.001
				LUAD	-0.338	< 0.001
				KIRC	-0.357	< 0.001
ZNF217	cg09228833	S_Shore	TSS200	UCEC	-0.278	< 0.001
	cg20979153	S_Shore	TSS200	UCEC	-0.282	< 0.001
ZNF445	cg18129786	S_Shore	TSS1500	LIHC	-0.282	< 0.001
ZNF512	cg01464985	N_Shore	TSS1500	UCEC	-0.490	< 0.001
ZNF560	cg18568589	S_Shore	TSS200	STAD	-0.347	< 0.001
				LUAD	-0.480	< 0.001
				LIHC	-0.256	< 0.001
				UCEC	-0.255	< 0.001
	cg05221167	S_Shore	TSS200	STAD	-0.316	< 0.001
				LUAD	-0.380	< 0.001
ZNF704	cg12077319	S_Shore; S_Shore	TSS200	STAD	-0.323	< 0.001
				LUAD	-0.270	< 0.001
ZNF781	cg03611452	CpG island	TSS200	STAD	-0.357	< 0.001
				LUAD	-0.389	< 0.001
				LIHC	-0.391	< 0.001
				KIRC	-0.432	< 0.001
				UCEC	-0.310	< 0.001
ZSCAN1	cg25537993	CpG island	TSS1500	STAD	-0.260	< 0.001
				LUAD	-0.447	< 0.001
				KIRC	-0.345	< 0.001
				UCEC	-0.410	< 0.001

ZSCAN18	cg14231297	CpG island	TSS200	STAD	-0.475	< 0.001
				KIRC	-0.336	< 0.001
				UCEC	-0.317	< 0.001

[a]Probe ID of the Infinium HumanMethylation 450 BeadChip. [b]N-Shelf: 2000 bp region 5' adjacent to N-Shore; N-Shore: 2000 bp region 5' adjacent to CpG island; S-Shore: 2000 bp region 3' adjacent to CpG island; S-Shelf: 2000 bp region 3' adjacent to S-Shore. [c]TSS200: 200bp upstream of the transcription start site (TSS); TSS1500: from 1500bp upstream of TSS to 200bp upstream of TSS. [d]KIRC: clear cell carcinoma of the kidney; LIHC: hepatocellular carcinoma of the liver; LUAD: adenocarcinomaof the lung; STAD: adenocarcinoma of the stomach; UCEC: endometrial carcinoma of the uterine corpus.

Supplementary Table S5 Correlations between the levels of mRNA expression and DNA methylation in the normal control liver tissue (C) samples (n=31 and n=36 for mRNA expression and DNA methylation quantification, respectively), non-cancerous liver tissue samples (N) (n=22) obtained from patients with non-alcoholic steatohepatitis-related hepatocellular carcinomas, and the corresponding cancerous tissue samples (T) (n=22).

				C (n=36)				N (n=22)				T (n=22)			
Gene	Probe ID[a]	Chrosome	Position[b]	Average mRNA expression levels (/GAPDH, mean±SD) (n=31)	Average DNA methylation levels (mean±SD) (n=36)	Correlation coefficient (r, n=31)	P[c] (n=31)	Average mRNA expression levels (/GAPDH, mean±SD)	Average DNA methylation levels (mean±SD)	Correlation coefficient (r)	P[c]	Average mRNA expression levels (/GAPDH, mean±SD)	Average DNA methylation levels (mean±SD)	Correlation coefficient (r)	P[c]
DCAF4L2	cg02310286	8	88886432	0.009±0.016	0.884±0.021	0.183	0.402	0.001±0.000	0.840±0.116	-0.043	0.850	0.331±0.546	0.387±0.251	-0.536	<u>0.010</u>
	cg17703554	8	88886339		0.805±0.023	0.017	0.939		0.789±0.090	-0.002	0.992		0.374±0.188	-0.435	<u>0.043</u>
	cg21181391	8	88886411		0.808±0.030	0.082	0.710		0.787±0.134	0.044	0.844		0.369±0.201	-0.497	<u>0.019</u>
CKLF	cg08287306	16	66585924	0.525±0.485	0.374±0.041	-0.065	0.730	0.328±0.097	0.308±0.061	0.088	0.696	0.984±0.686	0.163±0.064	-0.238	0.286
	cg08793208	16	66585449		0.504±0.055	-0.098	0.600		0.406±0.065	-0.018	0.935		0.243±0.108	0.0290	0.898
TRIM4	cg02756107	7	99517460	0.845±0.578	0.492±0.068	0.123	0.509	0.815±0.254	0.472±0.047	0.150	0.505	2.010±1.642	0.233±0.119	-0.562	<u>0.006</u>
	cg25591451	7	99517509		0.513±0.069	0.101	0.590		0.469±0.078	0.331	0.133		0.206±0.118	-0.675	<u>< 0.001</u>
PRC1	cg01407062	15	91538575	0.014±0.013	0.684±0.050	-0.081	0.666	0.023±0.021	0.589±0.055	0.047	0.027	0.161±0.200	0.253±0.140	-0.083	0.715
UBE2C	cg19197419	20	44441612	0.210±0.414	0.369±0.050	0.019	0.921	0.093±0.124	0.296±0.056	0.215	0.337	0.753±0.780	0.130±0.122	-0.019	0.934
TUBA1B	cg13709639	12	49526040	0.011±0.011	0.628±0.051	-0.208	0.261	0.016±0.0061	0.513±0.080	0.185	0.411	0.023±0.017	0.222±0.139	-0.171	0.448

[a]Probe+A8:R14 ID for the Infinium HumanMethylation450 BeadChip (Illumina).

[b]National Center for Biotechnology Information database (Genome Built 37).

[c]P -values of less than 0.05 were considered statistically significant and underlined when r was less than -0.3.

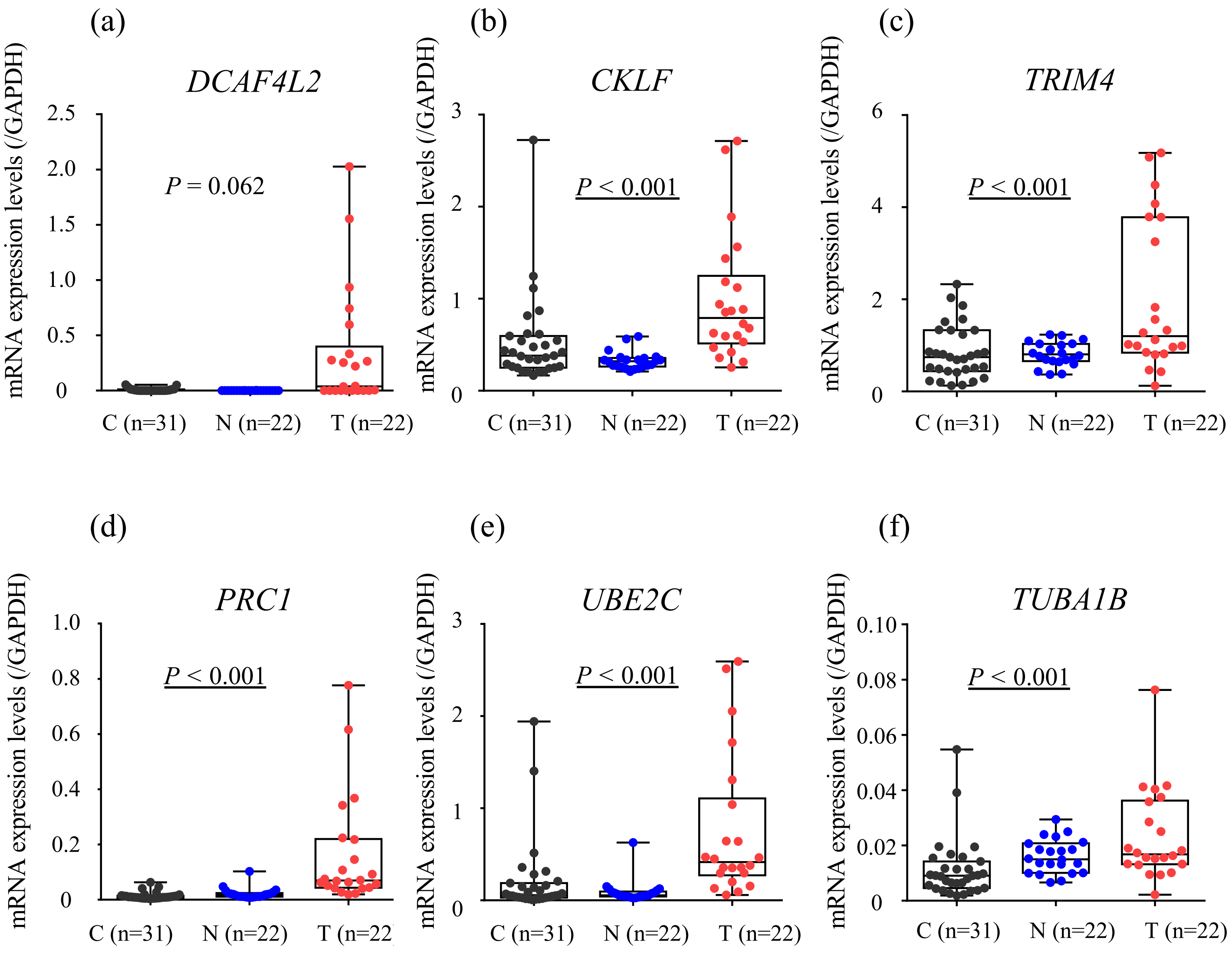

Supplementary Figure S1 Ordered differences in levels of mRNA expression for the *DCAF4L2* (a), *CKLF* (b), *TRIM4* (c), *PRC1* (d), *UBE2C* (e) and *TUBA1B* (f) genes in normal control liver tissue (C) (n=31) and non-cancerous liver tissue (N) (n=22) obtained from patients with non-alcoholic steatohepatitis-related hepatocellular carcinomas relative to the corresponding tumorous tissue (T) samples (n=22). *P* values by Jonckheere-Terpstra trend test are shown in each panel. *P* values of < 0.05 are underlined.

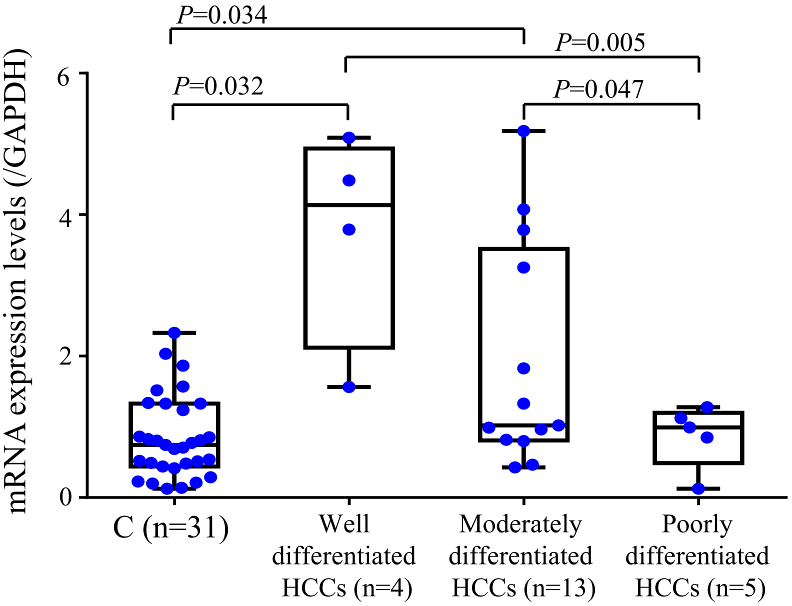

Supplementary Figure S2 Levels of mRNA expression for the *TRIM4* gene in samples of normal control liver tissue (C) (n=31) and tumorous tissue (T) (n=22). T samples were grouped on the basis of tumor differentiation. mRNA expression initially increased during the establishment of cancer, and thereafter decreased during the process of dedifferentiation from well to poorly differentiated HCC.

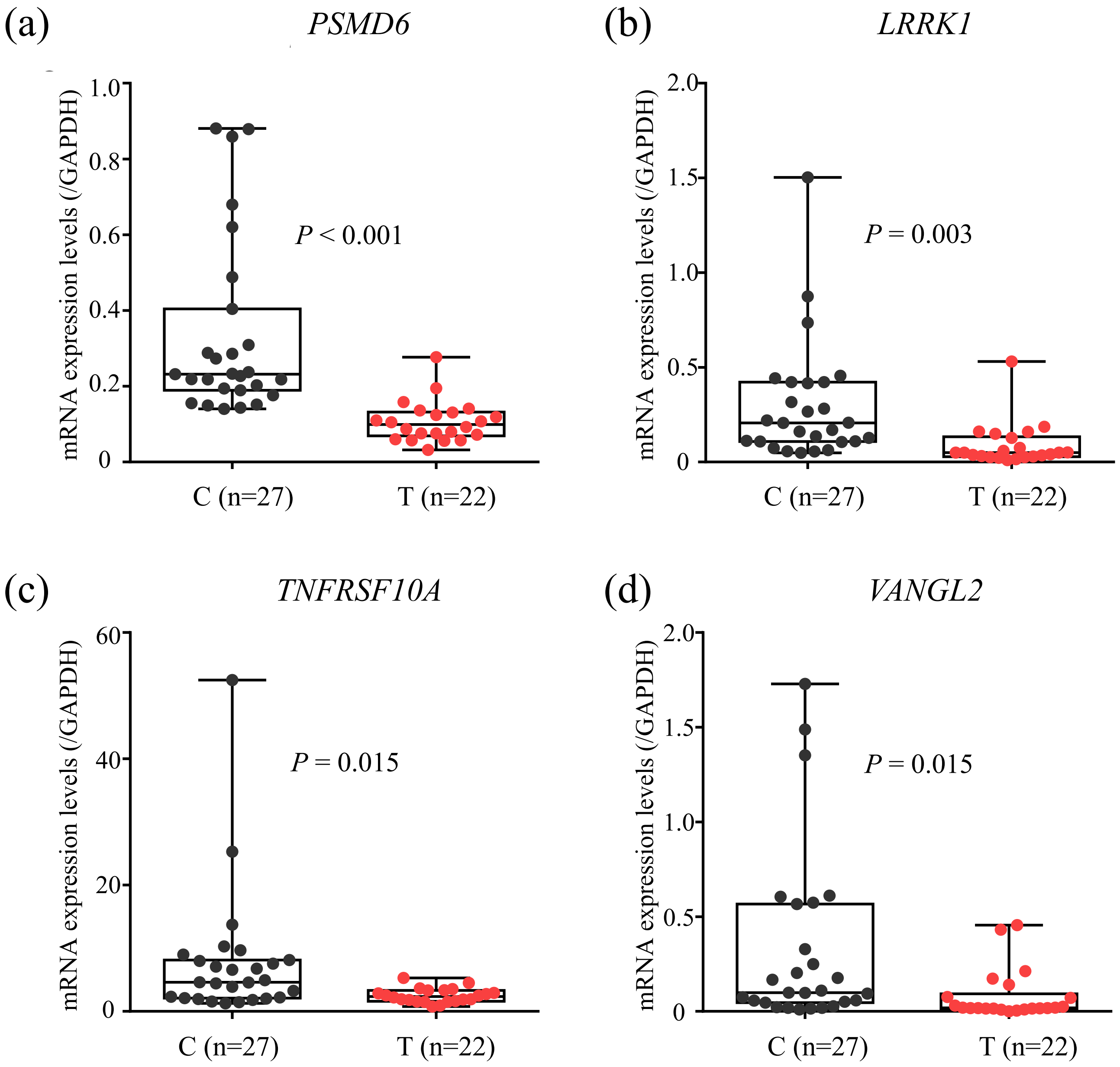

Supplementary Figure S3 Levels of mRNA expression for the representative genes involved in the NF-KB signaling and/or Wnt signaling pathways shown in Table 1, i.e. *PSMD6* (a), *LRRK1* (b), *TNFRSF10A* (c) and *VANGL2* (d), in samples of normal control liver tissue (C) and tumorous tissue (T). *P* values by Welch's t test are shown in each panel. Levels of mRNA expression for all 4 genes were significantly decreased in T samples (n=22) relative to C samples (n=27).
